# In Situ X‐Ray Techniques Unraveling Charge Distribution Induced by Halogen Bonds in Solvates of an Iodo‐Substituted Squaraine Dye

**DOI:** 10.1002/advs.202400661

**Published:** 2024-04-24

**Authors:** Xiaoyu Ye, Jonathan Gutenthaler‐Tietze, Ruoxuan Wu, Guomin Xia, Shidang Xu, Bin Liu, Yi‐Hung Chen, Konstantin Karaghiosoff

**Affiliations:** ^1^ The Institute for Advanced Studies (IAS) Wuhan University Wuhan 430072 China; ^2^ Department of Chemistry Ludwig‐Maximilians‐Universität München 81377 München Germany; ^3^ School of Biomedical Sciences and Engineering South China University of Technology Guangzhou 511442 China; ^4^ The Institute for Advanced Studies (IAS) Nanchang University Nanchang 330031 China; ^5^ Department of Chemical and Biomolecular Engineering National University of Singapore Singapore 117585 Singapore

**Keywords:** halogen bond, molecular electrostatic potential, SCSC, SCXRD, XANES

## Abstract

The importance of halogen bonds (XBs) in the regulation of material properties through a variation in the electrostatic potential of the halogen atom is not attracted much attention. Herein, this study utilizes in situ single crystal X‐ray diffraction and synchrotron‐based X‐ray techniques to investigate the cooling‐triggered irreversible single‐crystal‐to‐single‐crystal transformation of the DMF solvated iodo‐substituted squaraine dye (SQD‐I). Transformation is observed to be mediated by solvent‐involved XB formation and strengthening of electrostatic interaction between adjacent SQD‐I molecules. By immersing a DMF solvate in acetonitrile a solvent exchange without loss of long‐range ordering is observed. This is attributed to conservation of the molecular charge distribution of SQD‐I molecules during the process. The different solvates can be used in combination for temperature‐dependent image encryption. This work emphasizes the changes caused by XB formation to the electrostatic potentials of halogen containing molecules and their influence on material properties and presents the potential utility of XBs in the design of soft‐porous crystals and luminescent materials.

## Graphical abstract

Investigating the role of halogen bond in electrostatic potential variation, this study utilizes in situ single crystal X‐ray diffraction and synchrotron‐based X‐ray techniques to study the cooling‐triggered SCSC of DMF solvated crystals. These findings show solvent‐mediated XB formation and electrostatic interaction strengthening, crucial for crystal transformation. Furthermore, solvent exchange preserves long‐range ordering, offering potential for temperature‐dependent image encryption.

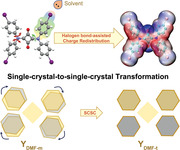

## 1 Introduction

The halogen bond (XB) has found extensive application in crystal engineering and material design.^[^ ^1^ ^]^ The polarizable halogen atoms have positive electrostatic potential regions (σ‐hole, δ+) and negative equatorial sides (negative belt, δ−), which provide opportunities to regulate electrostatic interactions.^[^ ^2^ ^]^ XBs, as a kind of charge‐transfer bonds, are defined to the transfer of negative charge from oxygen atoms, nitrogen atoms, or other Lewis bases to the halogen atom (Lewis acid), thus changing the electron density of halogen atoms.^[^ ^3^ ^]^ However, experimental evidence regarding the influence of XB on the charge distribution of halogen‐containing molecules is limited, except for computational studies.^[^ ^4^ ^]^

To prove this concept, the molecules under study should have sufficient flexibility. Additionally, cooperative donor‐π‐acceptor (D‐π‐A) systems, which in general are more susceptible to partial charge changes, can be beneficial.^[^ ^5^ ^]^ These molecules commonly display aggregation‐induced emission (AIE).^[^ ^6^ ^]^ The luminescent properties dependent on aggregation and conformation favor responsiveness tracking through optical signal.

**Figure 1:**
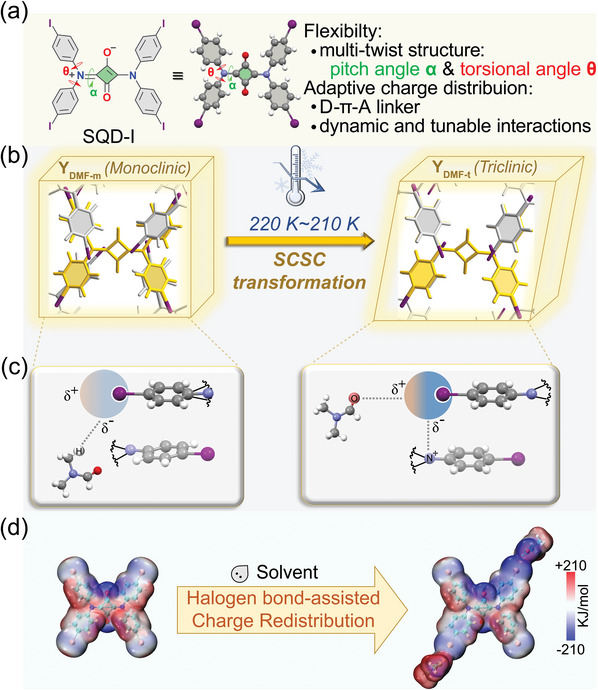
a) Structure and molecular properties of SQD‐I. b) Single‐crystal‐to‐single‐crystal (SCSC) transformation upon cooling. (Phenyl rings are colored in grey and yellow to distinguish between different layers, the yellow phenyl rings belong to the middle layer. c) The weak interactions change upon iodine during SCSC transformation. d) Solvents donate charge to iodine through halogen bonds, redistributing the charge within the entire molecule (SQD‐I).

**Scheme 1:**
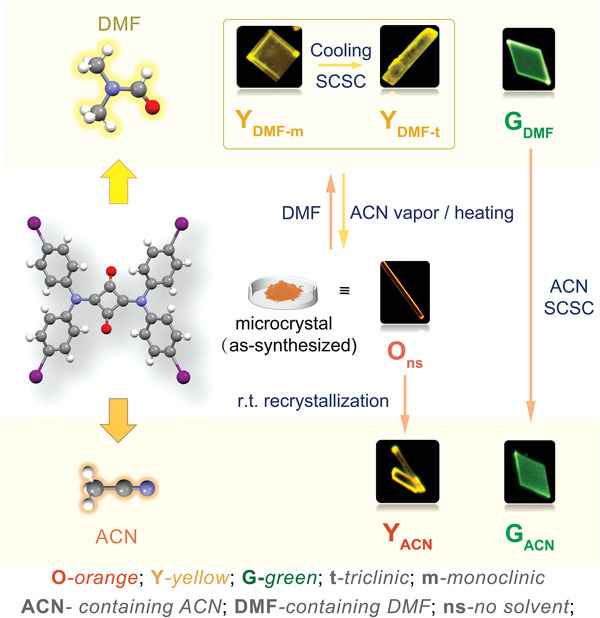
The abbreviations and transformations between crystals. (ACN: acetonitrile, DMF: *N, N*‐dimethyl formamide.).

Herein, based on the solvated and non‐solvated crystals of iodo‐substituted squaraine dye SQD‐I (an AIE luminogen) (**Figure** **1**a), we track and elucidate the crystal transformation and electronic properties by adopting in situ single‐crystal X‐ray diffraction (SCXRD) and synchrotron‐based X‐ray techniques.^[^ ^7^ ^]^ We provide insights into the solid‐state interaction of *N,N*‐dimethylformamide (DMF), and the iodo‐subsituent of SQD‐I during the cooling‐induced single‐crystal‐to‐single‐crystal transformation (SCSC) ^[^ ^8^ ^]^ from **Y_DMF‐m_** (monoclinic system) to **Y_DMF‐t_** (triclinic system) (Figure 1b). For the first time temperature‐dependent iodine electron density changes in crystals are quantified through synchrotron X‐ray experiments. Theoretical calculations of the electrostatic potential on iodine's negative belts also support the charge transfer from DMF to iodine via XB (Figure 1c). Moreover, the distinct responses of solvates (with different DMF and SQD‐I ratio) upon exposure to acetonitrile (ACN) proved the importance of XB's effect on stimuli‐responsive behavior (**Scheme** **1**). Solvents have a non‐negligible effect on charge distribution of halogen‐containing molecules via XBs, contributing to the materials’ design and paving the way for innovative approaches in solvent‐switchable soft crystals (Figure 1d).

## 2 Results and Discussion

### 2.1 Synthesis and Cooling‐Triggered Y_DMF‐m_ to Y_DMF‐t_ Single Crystal Transformation

The condensation of squaric acid with 4,4’‐diiododiphenylamine afforded SQD‐I as a bright orange microcrystalline powder (defined as **O_ns_**) in 85% yield.^[^ ^9^ ^]^ It underwent comprehensive characterization by ^1^H/^13^C NMR and high‐resolution mass spectrometry (Figures S1 and S2, Supporting Information).

Upon immersion in DMF, finely dispersed yellow‐green microcrystals were obtained within 30 min from the orange powders. Powder X‐ray diffraction (PXRD) patterns of these microcrystals aligned with those of the simulated yellow block single crystals (**Y_DMF‐m_**) (Figure S3, Supporting Information). Single crystals of **Y_DMF‐m_** were obtained by cooling an oversaturated DMF solution at room temperature. It crystallizes in the monoclinic space group *P2_1_/n*, with a SQD‐I:DMF molar ratio of 1:2 (**Table** **1**). In temperature‐controlled photoluminescence experiments (cooling to cryogenic temperature and rewarming), it was expected that the emission wavelength of **Y_DMF‐m_** would remain consistent at the same temperature. However, the fluorescence maximum of **Y_DMF‐m_** failed to revert post‐cooling to 100 K and rewarming (Figure S11, Supporting Information), suggesting changes in crystal packing or molecular conformation.

**Table 1:** The cell parameters of YDMF‐m and YDMF‐t (300 K).

	Y_DMF‐m_	Y_DMF‐t_
Crystal system	Monoclinic	Triclinic
Space group	*P2*_1_/n	*P*‐1
a/Å	11.0008(4)	10.1596(4)
b/Å	14.4283(5)	10.1596(4)
c/Å	12.4684(5)	10.7649(4)
α/°	90	115.826(4)
β/°	111.4500(10)	104.084(4)
γ/°	90	95.195(4)

**Figure 2:**
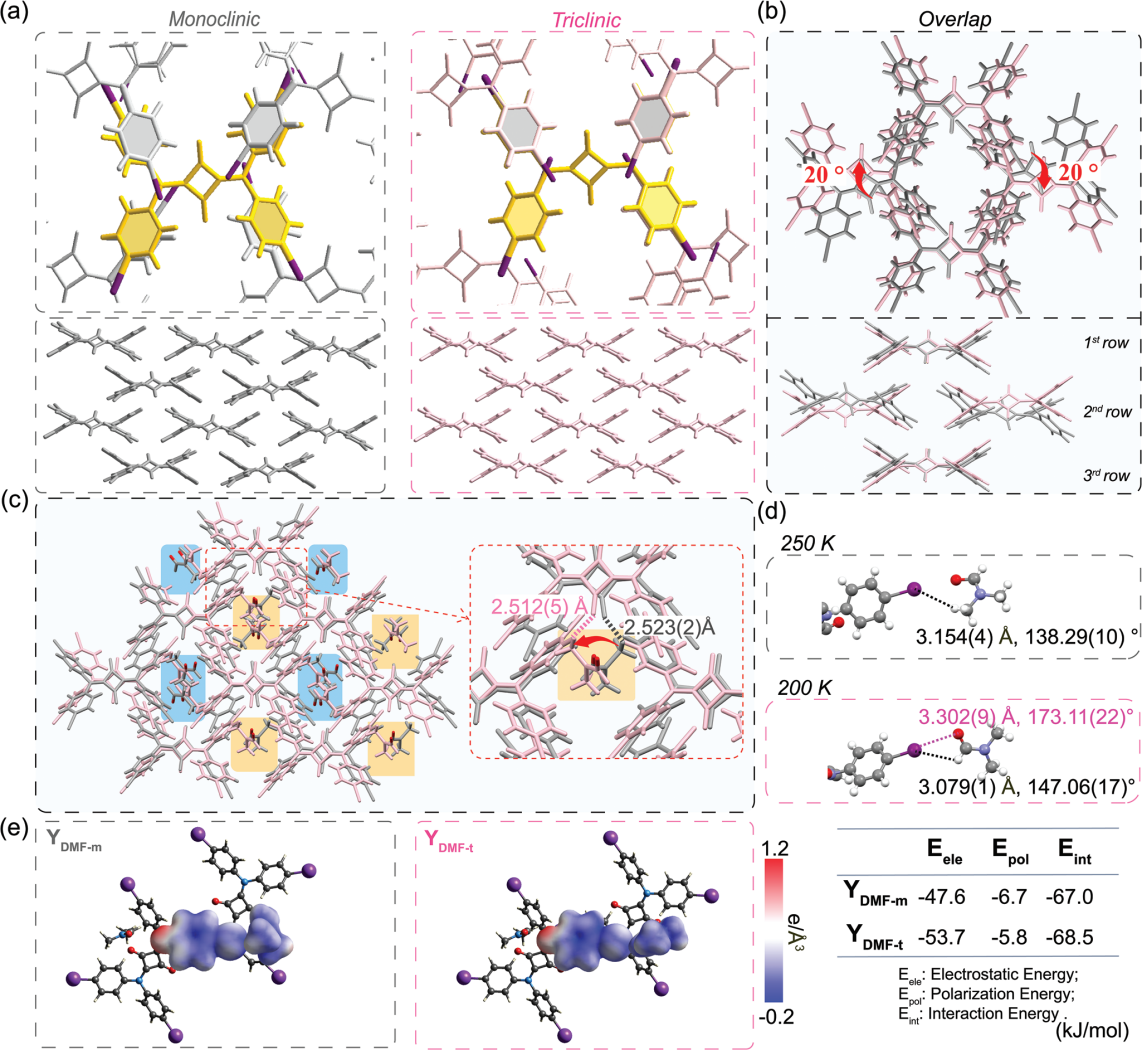
a) The top view and side view, and b) the overlap structure from top and side view of YDMF‐m‐250 K (gray) and Y DMF‐m‐200 K (pink) c) the overlay structure of YDMF‐m‐250 K (gray) and Y DMF‐m‐200 K (pink), and the DMF molecules on the orange background rotated, those on blue background kept the direction. d) HB/XB between I and DMF (right). e)The electron density isosurfaces of Y DMF‐m (300 K, right) and Y DMF‐t (300 K, left), and the table of interaction energies between SQD‐I molecules (kJ mol^−1^) (Color of electron density isosurfaces: red indicating positive potential and blue indicating negative potential. Calculation method: CE‐B3LYP/def2‐TZVP).

In situ variable‐temperature SCXRD revealed that **Y_DMF‐m_** underwent a single‐crystal‐to‐single‐crystal (SCSC) transformation to **Y_DMF‐t_** (triclinic system) at ≈210 K (Table S3, Supporting Information). Following the transformation, the phenyl rings in the upper and lower layers showed complete antiparallel stacking (**Figure** **2**a). While the squaraine dye in the first and third rows maintained almost identical orientation, the dye in the second row underwent rotation. (Figure 2b, bottom) Meanwhile, DMF (on an orange background) formed a hydrogen bond (HB, 2.523(2)/2.512(5) Å) with the oxocarbon anion of the aromatic four‐membered ring system, rotating together with squaraine (Figure 2c). By overlapping the two crystal structures in *Mercury*, we observed that half of the SQD‐I molecules underwent a rotation of ≈20° (Figure 2b, top). In **Y_DMF‐m_**, the DMF was engaged in C**H**_3(DMF)_•••**I** HB (3.154(4) Å, 138.29(10)°). In **Y_DMF‐t_**, the orthogonal C**H**O_(DMF)_ •••I (HB, 3.079(1) Å, 147.06(17)°) and I•••CH**O**_(DMF)_ (XB, 3.302(9) Å, 173.11(22)°) were identified (Figure 2d). The relationships among these two noncovalent interactions could be synergistic, where a HB enhanced the XB.^[^ ^10^ ^]^ Theoretical calculations suggested the antiparallel packing (**Y_DMF‐t_**) provided a more stable and robust assembly compared to **Y_DMF‐m_**, which suggests an energetic advantage of antiparallel π–π stacking (Figure 2e; Figures S37 and S40 and Scheme S2, Supporting Information).

### 2.2 Crystal Diffraction on Y_DMF‐t_ and XANES Experiment

**Figure 3:**
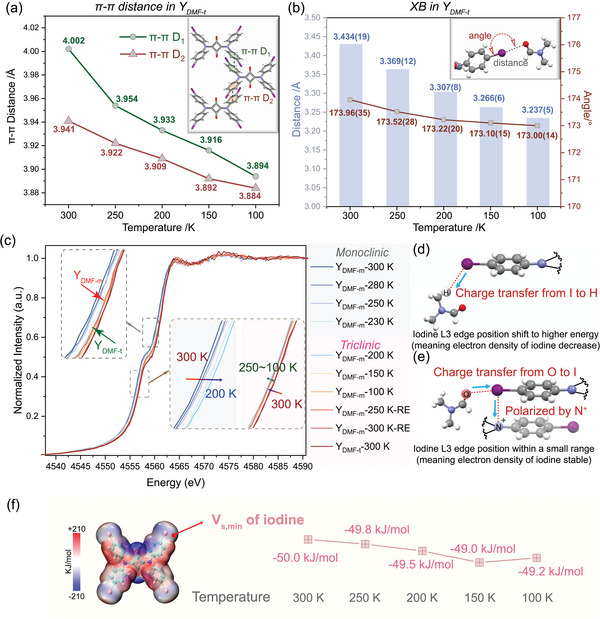
a) The temperature‐dependent π–π distance in **Y_DMF‐t_**. b) The temperature‐dependent XB distance and angle in **Y_DMF‐t_**. c) Normalized XANES spectra of **Y_DMF‐m_** and **Y_DMF‐t_** at the iodine L3‐edges. (Prefix is **Y_DMF‐m_** was the data collect with **Y_DMF‐m_** as the beginning sample. RE means the data collected during rewarming process. Prefix is **Y_DMF‐t_** was the data collect with **Y_DMF‐t_**.) The charge transfer in d)**Y_DMF‐m_** and e) **Y_DMF‐t_**. f) The surface electrostatic potential (V_s, min_) of iodine atom at different temperatures from the ESP results as shown in Figure S41 (Color of electron density isosurfaces: red indicating positive potential and blue indicating negative potential. Calculation method: B3LYP/DEF2TZVP).

Plank prismatic **Y_DMF‐t_** crystals could be crystallized from DMF solution at 298 K as well, belonging to triclinic space group *P‐1* (Table 1; Figures S12 and S13, Supporting Information). Temperature‐dependent SCXRD data revealed the shortening of both π–π and XB distances. (**Figure** **3**a,b) To further explore the changes in electron density of iodine upon cooling, we conducted synchrotron‐based X‐ray experiments. X‐ray absorption near edge structure (XANES) spectroscopy is suitable for identifying both the chemical environment and oxidation state of iodine across different temperatures, thus reflecting changes in iodine electron density.

Utilizing the **Y_DMF‐m_** microcrystals at 300 K as the starting point (confirmed with PXRD patterns, Figure S29, Supporting Information), the iodine L3‐edge spectra showed a consistent shift to higher energy as the temperature decreased. The shift progressed from 4557.76 eV (at 300 K) to 4557.81 eV (at 280 K), 4557.96 eV (at 250 K), and finally to 4558.19 eV (at 230 K) (Figure S30, Supporting Information). (Figure 3c) This trend indicated a potential increase in the formal charge of iodine atoms within the monoclinic system (**Y_DMF‐m_**) as the temperature decreases. Analysis of single‐crystal data suggested the shortened HB distance likely attributable to the changing of electron density around iodine (Figure 3d).

However, the same sample, following the SCSC transformation at 200 K (**Y_DMF‐t_**), exhibited a contrasting behavior in the L3 edge energy. The absorption edge showed a small shift to lower range (4558.65 to 4558.53 eV) (Figure 3c). The edge position did not return to the initial position (**Y_DMF‐m_**) after rewarming but was consistent with another **Y_DMF‐t_** sample at 300 K. According to the discussion above in Figure 3a,b, the shorter π–π distances indicated the enhancement of iodine polarization by N^+^ (SQD‐I), and the closer oxygen atom (DMF) replenished the iodine charge via XB σ‐hole simultaneously (Figure 3e). These two antagonistic effects resulted in the stability in edge energy in **Y_DMF‐t_**. Surface ESP analysis^[^ ^11^ ^]^ of iodine showed V_s,min_ values ranging between −45.606 and −46.610 kJ mol^−1^ (Figure 3f), further supporting that the electron density of iodine atoms remained nearly constant.

### 2.3 Revisiting the Significance of Solvent Molecules in the Charge Distribution of Halogen‐Containing Molecules

Based on the discussion above, it was realized that the solvent has a certain impact on the electrostatic potential of the halogen‐containing molecule. We continued to explore the solvent regulation of the charge distribution and the influence of the material properties which are underexplored in the past. SQD‐I encompasses π‐conjugated systems with *N*‐connected π‐units, characterized by a multi‐twist angle structure.^[^ ^12^ ^]^ Thus, the solvent‐induced modulation of the electron density at the terminal iodine atom through XB might consequently cause the redistribution of the charge of the entire molecule through the adjusting the torsion angles and packing patterns.

**Figure 4:**
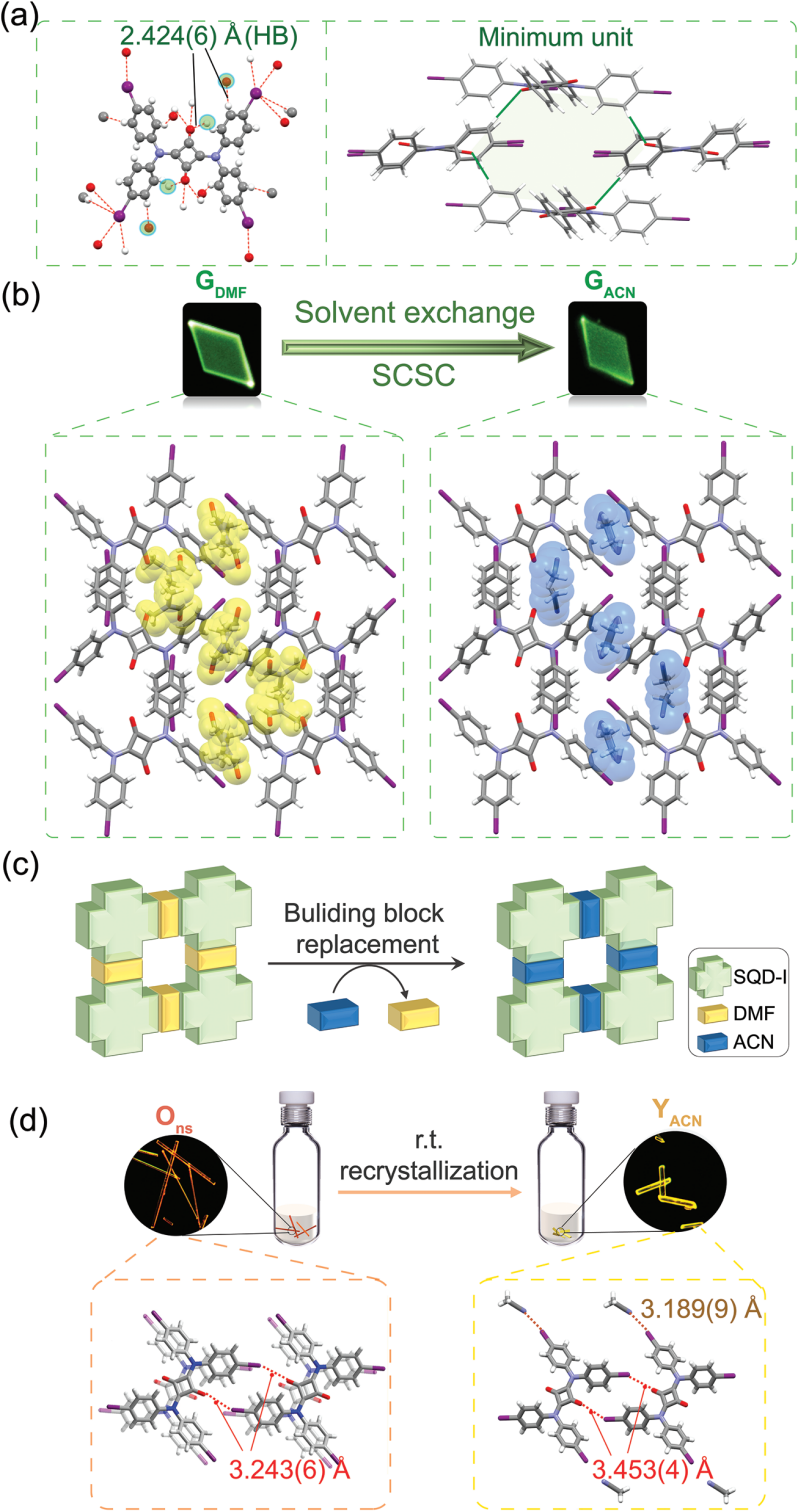
a) The HB between SQD‐I molecules and the minimum repeating unit built by SQD‐I through HBs. b) The SCSC transition from **G_DMF_** to **G_ACN_**, and crystal analysis. c) Solvents acted as a building block to support frameworks. d) The recrystallization process from **O_ns_** to **Y_ACN_** (top) and the XBs (bottom).

Here, vibrant green fluorescent rhombus‐shaped single crystals (**G_DMF_**) were produced by cooling the over‐saturated DMF solution rapidly at ≈230 K. (Keeping the oversaturated solution in an cooling bath of ethanol‐dry ice for 3 min followed by removing the ice bath leads to crystal growth. If the solution maintained in ice bath till all dyes precipitated, it resulted in crystals too small for SCXRD experiment or optical microscopy.) Crystal analysis revealed the existence of a kind of HB (2.424(6) Å) between SQD‐I molecules, which formed the minimum repeating unit through four HBs (**Figure** **4**a). Concurrently, the Hirshfeld surface analysis^[^ ^13^ ^]^ revealed that the short I···O interaction with DMF dominated all close contacts (Figure S34e, Supporting Information). The DMF molecules significantly contribute to the assembly of the host‐guest system as a building block. Traditional frameworks commonly rely on numerous and strong interactions among host molecules, which would otherwise lead to the collapse of the long‐range ordered arrangement upon guest exchange.^[^ ^14^ ^]^ On the contrary, soaking in ACN for 30 min allowed DMF to be replaced by ACN, resulting in a SCSC transformation of **G_DMF_** into **G_ACN_** (Figure 4b; Figure S14 and Video S1, Supporting Information.). As shown in Figure S41 (Supporting Information), the difference between the surface local minima values of iodine (I) and oxygen (O) is found to be very similar. For **G_DMF_**, the ΔV_s,min_ are 119.1 and 134.5 kJ mol^−1^ for I and O, respectively, while for **G_ACN_**, ΔV_s,min_ are 119.2 and 133.5 kJ mol^−1^. This supported the idea that introduction of new guest molecules (ACN) maintains the original charge distribution, thereby preserving the twist angle and favoring the stabilization of crystal packing (Table S2, Supporting Information). This breathing structural transformation involved shrinking of the void space with the change of solvent molecules, presenting a rare example of guest exchange based on such a loose skeleton (Figure S42, Supporting Information) (Figure 4c).

On the other hand, orange needle‐like single crystals (**O_ns,_** *non‐solvated*) of SQD‐I crystallized at ≈330 K with simulated PXRD patterns matching the synthesized orange powder (Figure S4b, Supporting Information), indicating the same tendency to establish the **O_ns_** packing at high temperatures (Figure S9c, Supporting Information). Crystal analysis of **O_ns_** revealed that the SQD‐I molecules were arranged in a 2D sheet held together by both XBs (3.243(6) Å) and HBs (2.669(7) Å and 2.630(7) Å) (Figure 4d; Figure S17, Supporting Information). Eventually, these 2D sheets further assembled into the third dimension through weak van der Waals interactions (Figure S31, Supporting Information).

In **O_ns_**, two of the four iodine groups have no significant intermolecular interactions. Recrystallization of yellow stick‐shaped crystals (**Y_ACN_**) can be observed after soaking **O_ns_** in ACN for one week. In **Y_ACN_**, the previously unoccupied iodine atoms formed XBs with ACN (3.189(9)Å) (Figure 4d, right). This resulted in a reduced pitch angle (α), disrupting of conjugation and enhancing the strength of XB donors by activating the σ‐hole, facilitating the formation of four sets of XBs. However, exposing **Y_DMF‐m_** and **Y_DMF‐t_** crystals to ACN vapors led to collapse, transforming into the **O_ns_** stacking mode (Figures S9 and S10, Supporting Information).

Typically, the focus on halogen atom revolves around the formation of weak interactions, neglecting the possible impact of XBs on the overall charge distribution and molecular conjugation, which has the potential to affect the material's stimulus response behavior. In the case of a D‐π‐A molecule with multiple rotors, such as SQD‐I, the initial formations of the XBs participate its charge distribution. Introducing a new XB acceptor might change this distribution, inevitably influencing the original halogen bond. In the solvates of SQD‐I, the presence of initially unoccupied iodo substituents means the molecules change their torsion angles during the interaction with ACN, leading to rapid collapse or slow recrystallization, as observed in **O_ns_**, **Y_DMF‐m_**, and **Y_DMF‐t_**. However, in **G_DMF_** all iodo substituents are already engaged in XBs, thus a sequential substitution of the DMF molecules by acetonitrile leaving the crystal skeleton intact seems feasible.

**Figure 5:**
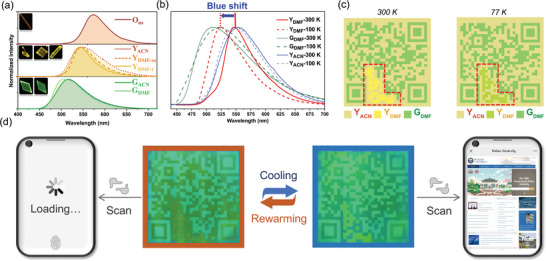
a) PL spectra at 300 K. b) The PL spectra of three solvates at 300 K/100 K. c) The designed pattern of the information encryption model fabricated by Y_ACN_, Y_DMF_, G_DMF,_ and the passage diagram at different temperature. d) The photographs of the designed QR code (printed as Figure 5c showing) to showcase how to read‐out by a smartphone after cooling (excitation: 365nm UV lamp).

Structural analysis of **O_ns_**, **Y_DMF‐m_**, **Y_DMF‐t_**, and **G_DMF_** revealed that a larger pitch angle (α) favors electron conjugation between nitrogen and the aromatic ring, resulting in a redder emission. A smaller pitch angle (α) decreases the potential for stable conjugation, resulting in a blueshift in fluorescence accompanied by an increased torsional angle (θ) (**Figure** **5**a; Table S2, Supporting Information).^[^ ^15^ ^]^ Moreover, these solvates exhibited distinct redshift and blueshift behaviors when the temperature decreased (Figure S11, Supporting Information). In crystals where SQD‐I forms two XBs (**O_ns_**, **Y_DMF‐m_**, **Y_DMF‐t_**, as well as **G_ACN_**), they exhibit blue shifts with decreasing temperature. However, SQD‐I with four XBs did not shift (**Y_ACN_**) or even showed a slight red shift (**G_DMF_**) upon cooling. The intermolecular charge transfer upon cooling in crystals with all iodine atoms occupied differs from those with only two occupied, reflected in distinct temperature‐dependent luminescence behaviors. **G_DMF_** and **Y_ACN_** powders exhibit minimal changes in their maximum emission wavelengths upon cooling, while **Y_DMF‐m_** shift from yellow (548 nm) to green (510 nm) (Figure 5b). It forms the basis for an encryption system composed of **G_DMF_**, **Y_ACN_**, and **Y_DMF‐m_** powders. By deposing these three crystals powder on the corresponding position (Figure S15, Supporting Information), we designed a code can be read under UV‐irradiation at low temperature (100 K or lower) (Figure 5c). The QR code lost its information in the absence of refrigeration and led to a scanning error. After cooling with liquid nitrogen (≈77 K), the emission of the **Y_DMF‐m_** region would blue‐shift, completing the QR code information with the emission band similar to green in the **G_DMF_** region. Thus, the code could be scanned and linked to the Wuhan University website (Figure 5d).

## 3 Conclusion

In summary, SQD‐I contains iodine, nitrogen, and oxygen atoms, as well as D‐π‐A rotators, achieving various packing conformations. Experimental results and theoretical calculation clearly demonstrated XB‐assisted charge redistribution, which were generally not explicitly considered. This concept could be observed in the examples of the recrystallization process from non‐solvated **O_ns_** to acetonitrile solvated **Y_ACN_**, and the SCSC phase transition from **Y_DMF‐m_** to **Y_DMF‐t_** upon cooling. The SCSC transformation from **G_DMF_** to **G_ACN_** also supported that the solvent can be exchanged without losing the integrity of the framework by maintaining the charge distribution via XBs. The temperature‐dependent luminescence properties also showed great potential for image encryption. These discoveries broaden the horizons concerning stimuli‐responsive materials in solvates, shedding light on a perspective that is easily underestimated in the exploration of XB for crystal engineering. Moreover, this unexpected irreversible SCSC transformation (**Y_DMF‐m_** to **Y_DMF‐t_**) upon cooling emphasizes that scientists should be more rigorous about the temperature used in structural characterization and other physical property tests, rather than focusing solely on the selection of cryogenic conditions due to the improved Bragg reflections in SCXRD experiments.^[^ ^16^ ^]^

## Conflict of Interest

The authors declare no conflict of interest.

## Supporting information

Supporting Information

Supplemental Video 1

## Data Availability

The data that support the findings of this study are available in the supplementary material of this article.
