# Supplementary material for: In Situ X‐Ray Techniques Unraveling Charge Distribution Induced by Halogen Bonds in Solvates of an Iodo‐Substituted Squaraine Dye
Source: Adv Sci (Weinh). 2024 Apr 24;11(25):2400661. doi: 10.1002/advs.202400661 (PMC11220701; doi:10.1002/advs.202400661)
Supplement: Supplementary file 1 — Supporting Information [file ADVS-11-2400661-s001.pdf]

ADVANCED SCIENCE
Open Access

Supporting Information

for *Adv. Sci.*, DOI 10.1002/advs.202400661

In Situ X-Ray Techniques Unraveling Charge Distribution Induced by Halogen Bonds in Solvates of an Iodo-Substituted Squaraine Dye

Xiaoyu Ye, Jonathan Gutenthaler-Tietze, Ruoxuan Wu, Guomin Xia, Shidang Xu*, Bin Liu*, Yi-Hung Chen* and Konstantin Karaghiosoff*

Supporting Information

In situ X-ray Techniques Unraveling Charge Distribution Induced by Halogen Bonds in Solvates of an Iodo-Substituted Squaraine Dye

Xiaoyu Ye,[1] Jonathan Gutenthaler-Tietze,[2] Ruoxuan Wu,[4] Guomin Xia,*[3] Shidang Xu,*[4] Bin Liu,*[5] Yi-Hung Chen,*[1] Konstantin Karaghiosoff [2]

Affiliations:

[1] The Institute for Advanced Studies (IAS), Wuhan University, Wuhan 430072, China.

[2] Department of Chemistry Ludwig-Maximilians Universität München, München 81377, Germany.

[3] The Institute for Advanced Studies (IAS), Nanchang University, Nanchang 330031, China

[4] School of Biomedical Sciences and Engineering, South China University of Technology, Guangdong 510641, China.

[5] Department of Chemical & Biomolecular Engineering, National University of Singapore, 119077, Singapore.

*Corresponding authors. Email: guominxia@ncu.edu.cn (G.X.); xusd@scut.edu.cn (S.X.); cheliub@nus.edu.sg (B.L.); yihungchen@whu.edu.cn (Y.-H.C.).

Content

General Information **3**
Method 3
Synthesis and characterization 4
Crystal physical data **6**
Crystal structure analysis **14**
XANES experiment **21**
Calculations **20**
RDG Analysis 23
Hirshfeld Surface Analysis 25
Frontier orbitals calculations 27
Electrostatic potential (ESP) Analysis 29
Void Space Analysis 33
Crystal data and structure refinement table **34**
References **42**

I. General Information.

i. Method.

(1) Materials

Squaric acid (3,4-Dihydroxy-3-cyclobutene-1,2-dione, 98%) and DPA-I [bis(4-iodophenyl)amine, 95%] were purchased from Bidepharm and used without further purification. All solvents (toluene, *n*-butanol, DMF, ACN) were purchased from Sinopharm.

(2) Instruments

^{1}H and ^{13}C NMR spectra were recorded on a Bruker AVANCE NEO 600 MHz or Bruker AVANCE NEO 800 MHz using tetramethylsilane as the internal standard.

High-resolution mass spectrum (HRMS) was recorded on Thermo Fisher Orbitrap Elite mass spectrometer.

The single-crystal X-ray diffraction data were conducted on a Rigaku XtaLAB Synergy Custom (Cu Kα (λ = 1.54178 Å)) or Bruker D8 Venture X-ray diffractometer (Mo Kα (λ = 0.71073 Å)).

Powder X-ray diffraction (PXRD) experiments were measured with Rigaku SmartLab SE.

Scanning electron microscope (SEM) experiments were measured with TESCAN VEGA Compact.

UV-visible absorption spectra were recorded on a Lambda 750 spectrophotometer. Photoluminescence (PL) spectra were recorded on a Horiba FluoroMax-4 luminescence spectrometer.

The absolute PL quantum efficiencies (Φ_{PL}) were determined using a Horiba FL-3018 Integrating Sphere.

The temperature-dependent lifte measurement was performed on Horiba QuantaMaster 8000, equipped with DeltaDiode TCSPC Pulsed Sources (390 nm) or SpectraLED LED Phosphorescence Light Sources (390nm).

(3) Crystallographic Information

Data collections were performed at 300 K, 250 K, 200 K, 150 K, 100 K on a XtaLAB Pro: Kappa single diffractometer using Mo Kα (λ = 0.71073 Å) or Cu Kα (λ = 1.54178 Å). Using Olex2, the structures were solved with Superflip solution program[17] using Charge Flipping or SHELXT 2018/2 (Sheldrick, 2018) solution program[18] using Direct Methods and refined with the ShelXL refinement package[19] using Least Squares minimization. Refinement was performed on F^2 anisotropically for all the non-hydrogen atoms by the full-matrix least-squares method. The hydrogen atoms were placed at the calculated positions and were included in the structure calculation without further refinement of the parameters. In some crystal structures, the commands DELU, SIMU and ISOR were applied to restrain the disorders of the molecules. Crystallographic data have been deposited with the Cambridge Crystallographic Data Centre as supplementary publication no. CCDC 2277085-2277103. The high extinction coefficients can be explained by the use of Cu Kα radiation in combination with iodine containing single crystals. In turn, this is most likely responsible for higher than usual amounts of residual electron density in close proximity to the iodine atoms in the crystal structures.

The crystal structure analysis was performed with **Mercury 2022.3.0**, including measurement, structure overlay and voids calculations.

ii. Synthesis and characterization.

Scheme S1. Synthesis of SQD-I.

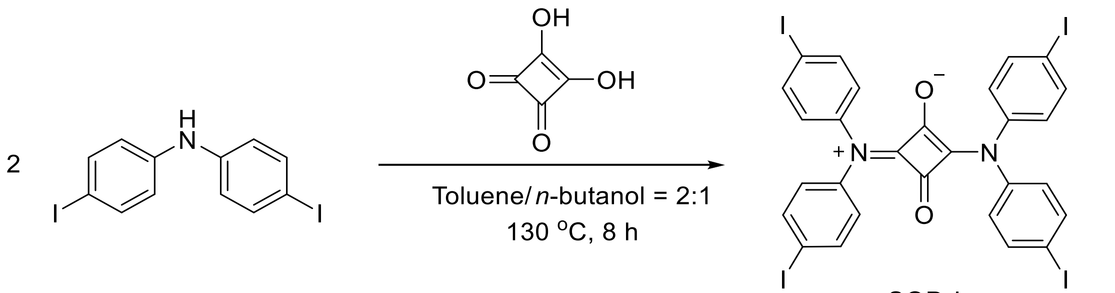

DPA-I SQD-I

2-(Bis(4-iodophenyl)amino)-4-(bis(4-iodophenyl)iminio)-3-oxocyclobut-1-en-1-olate (SQD-I): To a solution of squaric acid (113 mg, 1 mmol), and bis(4-iodophenyl)amine (DPA-I) (1.0 g, 2.375 mmol) in toluene/*n*-butanol (2:1 v/v, 6 ml) was degassed (three times) and was refluxed for 8 h with a Dean-Stark apparatus. After the reaction was completed, the reaction mixture was filtered and the residue was washed twice with methanol to give pure SQD-I (790 mg, 85.6%). **^{1}H NMR** (DMF-d$_7$, 600 MHz), δ (ppm): 7.21(d, 4H, J = 8.8 Hz, Ar-H), 7.86 (d, 4H, J = 8.8 Hz, Ar-H). **^{13}C NMR** (DMF-d$_7$, 201 MHz), δ (ppm): 181.63, 168.00, 140.96, 139.03, 128.68, 93.75. **HRMS (ESI)**, m/z: calcd. for $C_{28}H_{16}I_4N_2O_2$ $[M+H]^+$ 920.74633, found: 920.74578 as shown in Figure S1-S2.

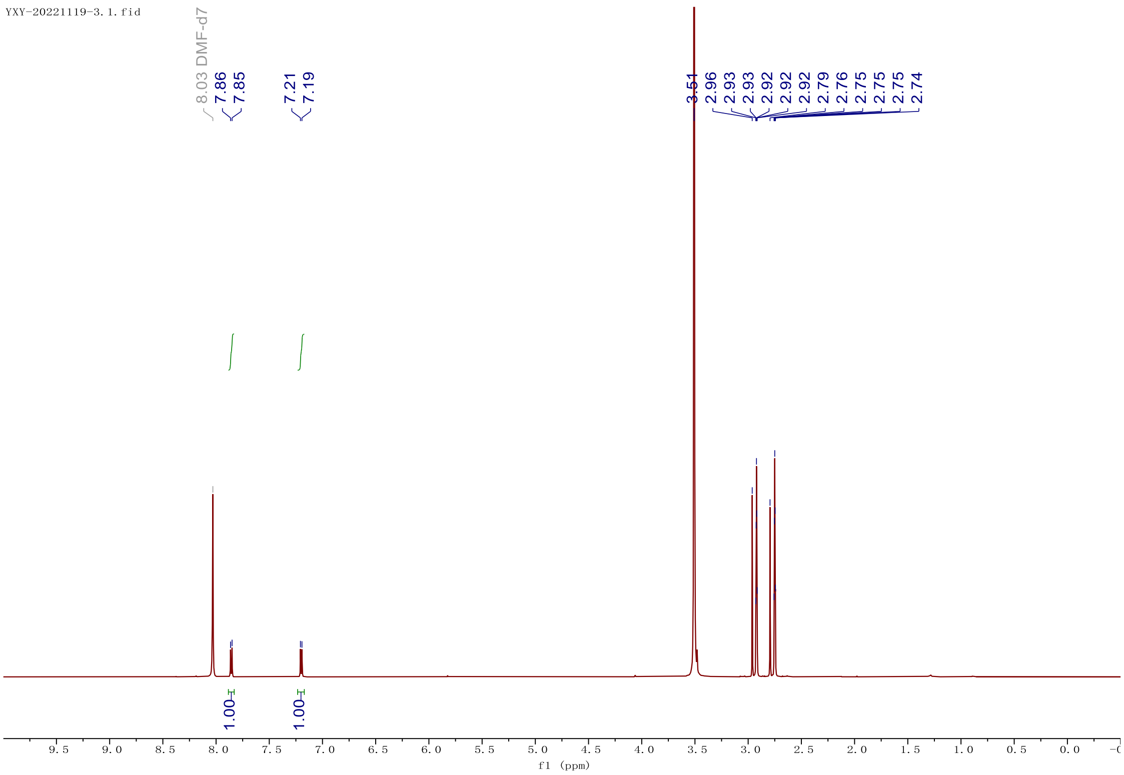

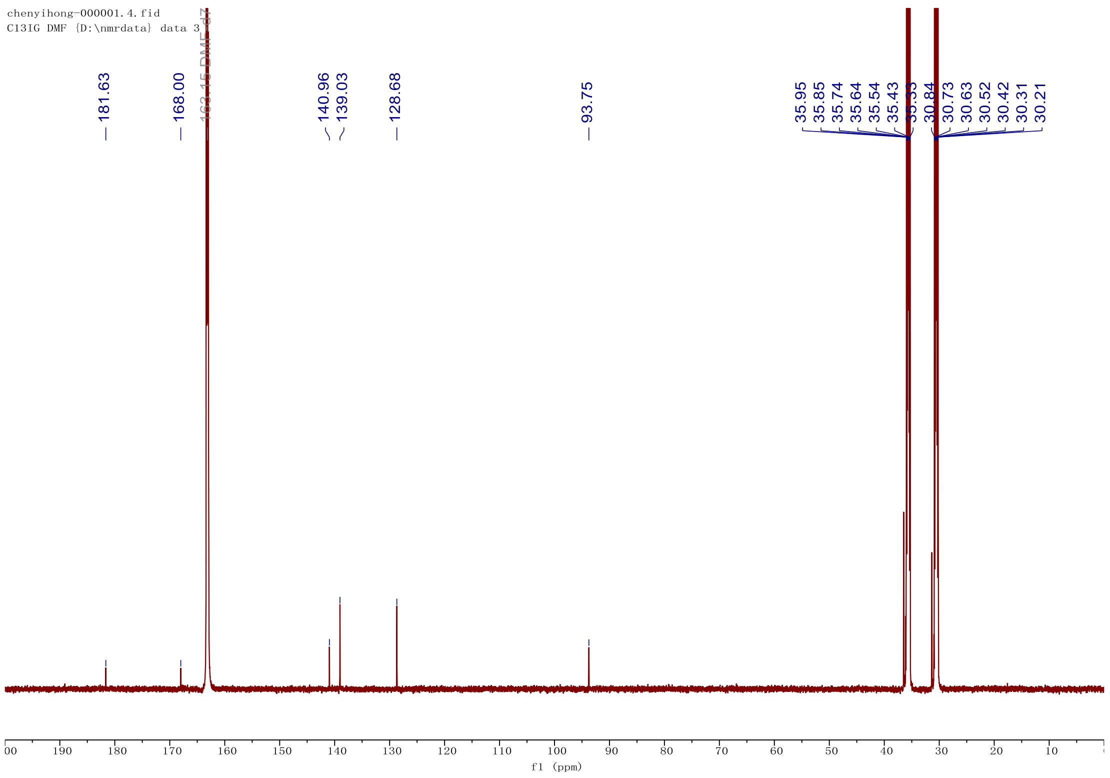

Figure S1. ^{1}H NMR and ^{13}C NMR spectra of SQD-I.

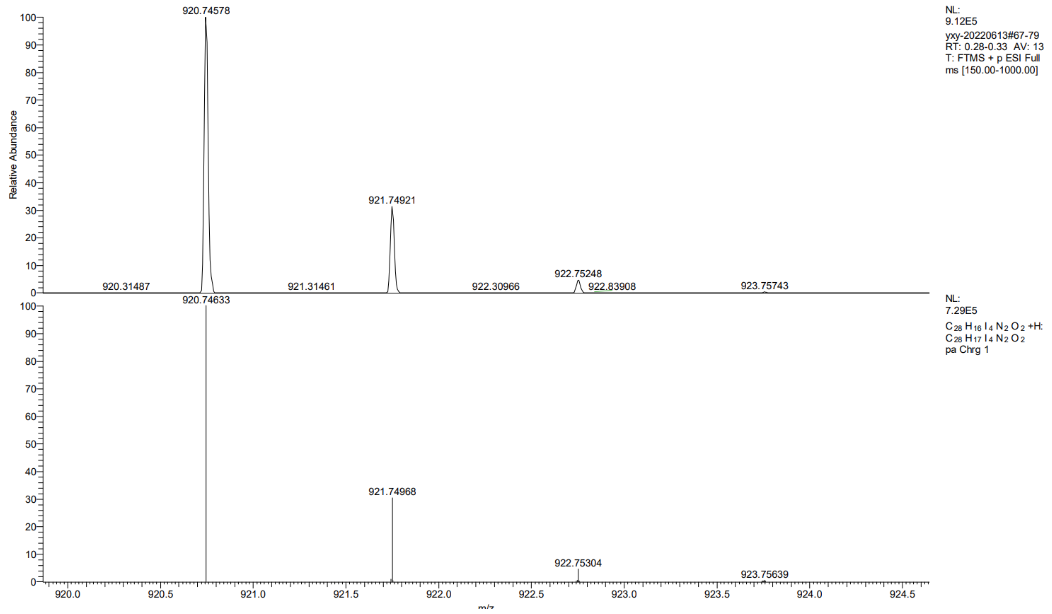

Figure S2. High-resolution mass spectrum (HR-MS) of SQD-I.

II. Crystal physical data.

Conditions for single crystal growth:

O$_{ns}$ /Y$_{ACN}$: The orange needle-like crystals (**O$_{ns}$**) could be obtained from oversaturated solution of SQD-I (2 mg) in ACN (30 mL) at 60 °C in a pressure-resistant flask for 1 day. The remaining solution and orange crystal was held at room temperature over 2-5 days, the orange crystal will turn into yellowish stick-like **Y$_{ACN}$** gradually. (Here we suggest to use the pressure-resistant flask to prepare **O$_{ns}$** because of the poor solubility of SQD-I in acetonitrile and low boiling point of ACN. The use of pressure-resistant flask can help to dissolve more SQD-I at higher temperature and crystallize at 60 °C.)

Y$_{DMF\text{-}m}$/Y$_{DMF\text{-}t}$: To a solution of SQD-I (70 mg) in DMF (10 mL) in an Erlenmeyer flask was heated up (120-130 °C) until it dissolved completely. The **Y$_{DMF\text{-}m}$** (yellowish blocks) and **Y$_{DMF\text{-}t}$** (stick-like crystal) were obtained when the solution returns to room temperature gradually.

G$_{DMF}$: To a solution of SQD-I (30 mg) in DMF (8 mL) in an Erlenmeyer flask was heated up till dissolved completely and the flask was transferred to -65 °C cooling bath for about 3 minutes. The greenish-flaky rhombohedral crystals could be obtained overnight. (We suggest to keep the oversaturated solution in an ice bath of ethanol-dry ice for 3 min to get seed crystals and remove the ice bath to grow crystal bigger. Otherwise, if stay in ice bath until all crystals precipitate, the crystals would be too small to SCXRD experiment or optical microscopy observation.)

G$_{ACN}$: Immersing G$_{DMF}$ single crystal in ACN for 30 minutes completed the single crystal transformation. It was recognized that the optical waveguide became weakened from **G$_{DMF}$** to **G$_{ACN}$**.

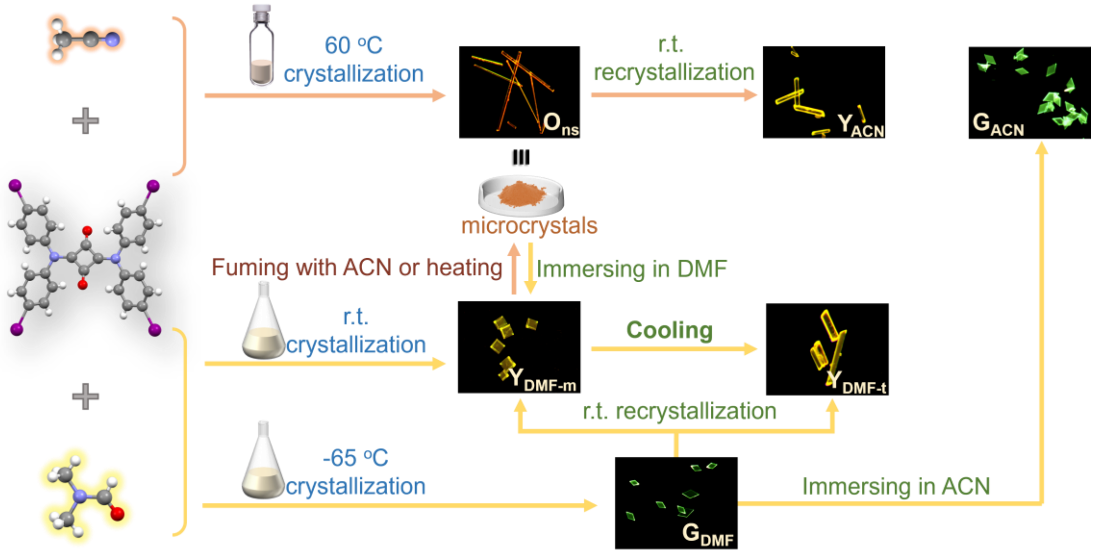

Figure S3. The conditions of single crystal preparation and crystal transformations.

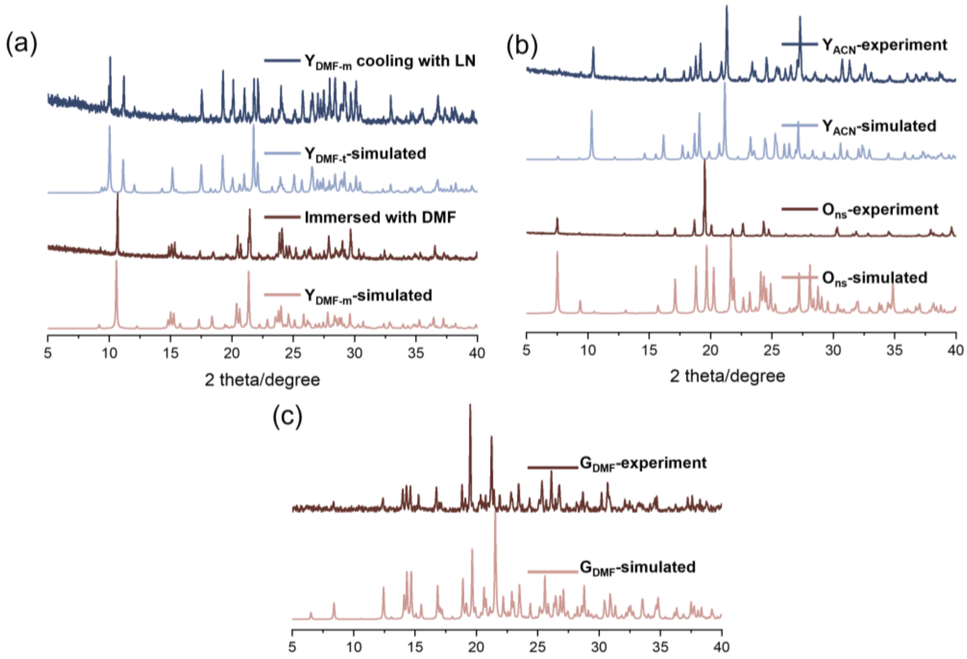

Figure S4. Powder X-ray diffraction (PXRD) patterns. (a) The PXRD patterns of $\mathbf{Y_{DMF\text{-}m}}$ and $\mathbf{Y_{DMF\text{-}t}}$. [The $\mathbf{Y_{DMF\text{-}m}}$ powder sample treated with liquid nitrogen (LN) could be consisted with $\mathbf{Y_{DMF\text{-}t}}$-simulated patterns.] (b) The PXRD patterns of $\mathbf{Y_{ACN}}$ and $\mathbf{O_{ns}}$. (c) The PXRD patterns of $\mathbf{G_{DMF}}$.

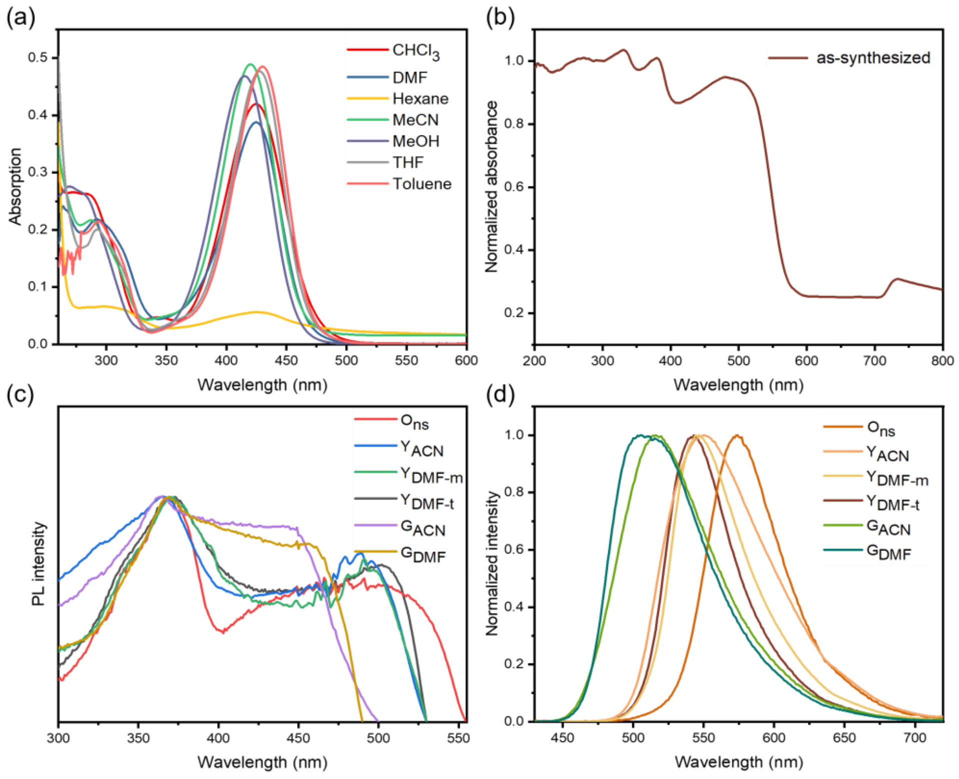

Figure S5. (a) Solution absorption spectra (Concentration: 10^{-5} M) in different solvents. (b) Normalized solid-state absorption spectra of SQD-I (freshly prepared). (c) The

excitation spectra (monitored emission wavelength is the corresponding maximum emission wavelength) and (d) emission spectra of six crystals (λ_{ex} = 365 nm). (All data were collected at room temperature).

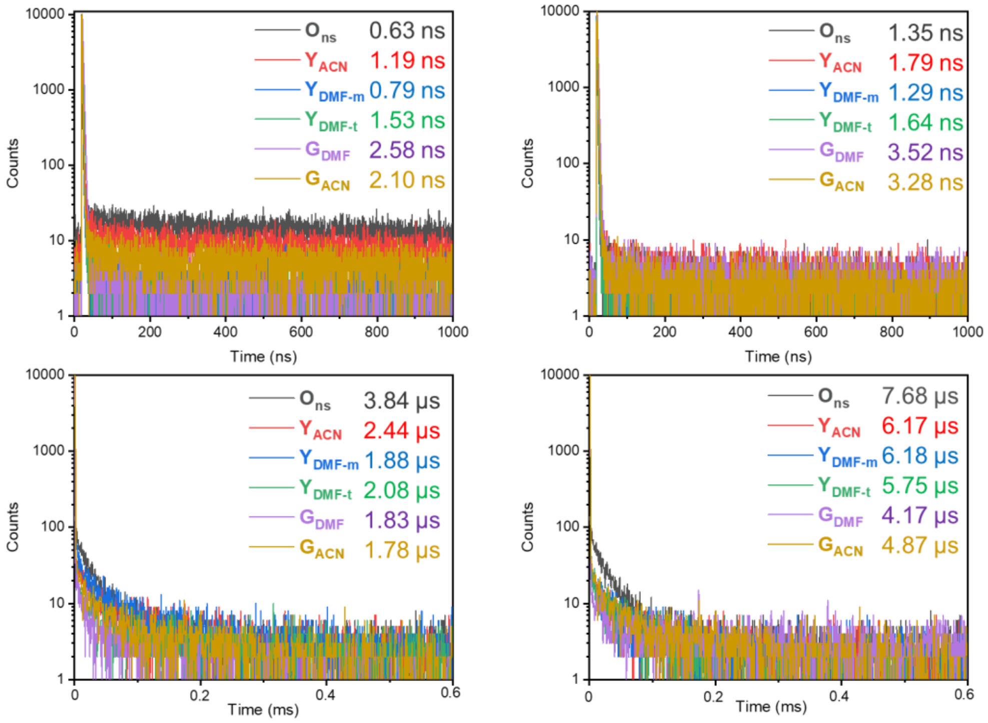

Figure S6. The PL decay carves and corresponding lifetime at (a) at 300 K (b) at 100 K (c) at 300 K (d) at 100 K. (Adopted Horiba light source: a,b: DeltaDiode TCSPC Pulsed Sources,390 nm ; c,d: SpectraLED LED Phosphorescence Light Sources, 390nm)

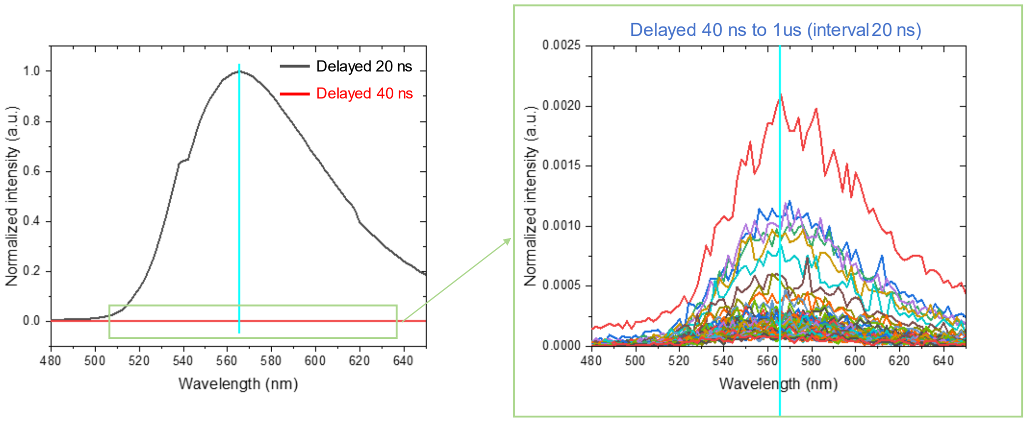

Figure S7. Time-resolved emission spectra upon 365 nm excitation with delay of the **O_{ns}** crystal.

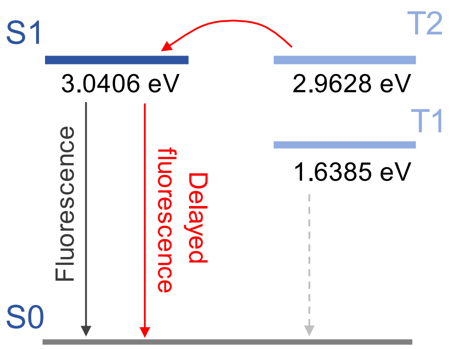

Figure S8. The time-dependent density functional theory (TD-DFT) calculated energy levels of singlet state(S1) and triplet states (Tn) of SQD-I monomer. (performed with Gaussian 16[29] with the B3LYP functional and the DEF2TZVP basis set[11], using $\mathbf{O_{ns}}$ crystal cif data, CCDC number: 2277097.)

The observed nanosecond lifetimes ranged from 0.63 to 2.58 ns at 300 K (Fig. S6a) and 1.29 to 3.52 ns at 100 K (Fig. S6b). We did also observe microsecond lifetimes which were identified in the range of 1.78 to 3.84 μs at 300 K (Fig. S6c) and 4.17 to 7.68 μs at 100 K (Fig. S6d).

Furthermore, based on time-resolved emission spectra (λ_{ex} = 365 nm) recorded for $\mathbf{O_{ns}}$ with delayed times ranging from 40 ns to 1 μs (Fig. S7), the spectra revealed that the unchanged maximum emission wavelength, which indicate the possibility of delayed fluorescence.

According to DFT calculation (Fig. S8), the observed singlet-triplet energy gap (ΔE_{ST}) is about 0.0778 eV, unveiling the potential for reverse intersystem crossing from triplet states to excited singlet states. This might be responsible for delayed fluorescence.

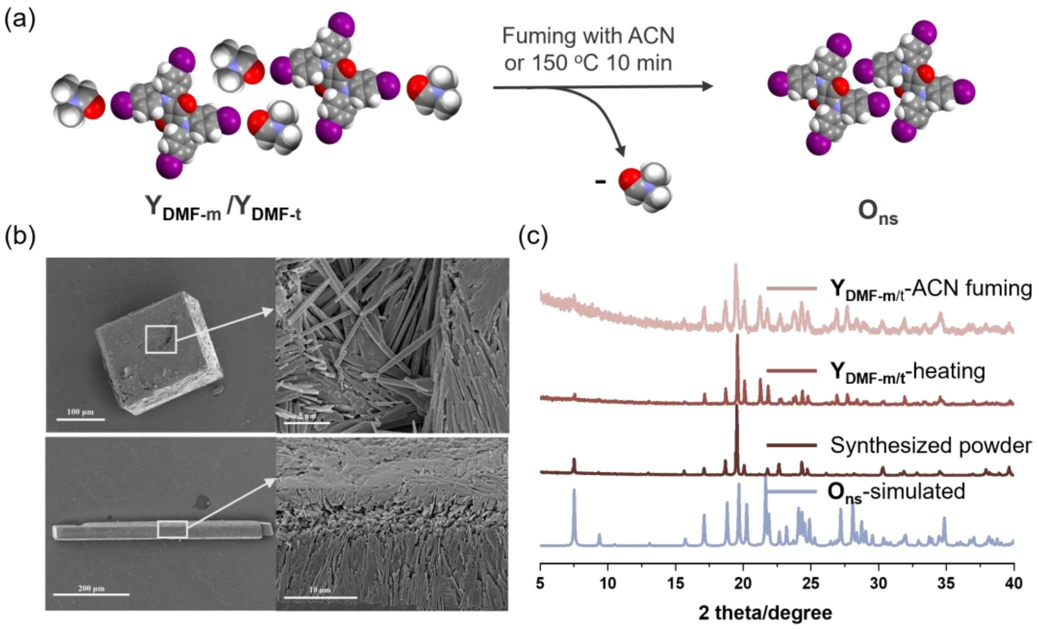

Figure S9 (a) **Y** $_{\mathbf{DMF\text{-}m}}$/**Y** $_{\mathbf{DMF\text{-}t}}$ released the DMF solvent after fuming with ACN or heating

for 10 min at 150 °C afforded the **O$_{ns}$** packing mode. (b) The scanning electron microscope (SEM) photographs after fuming with ACN (upper: **Y $_{DMF-m}$**, lower: **Y $_{DMF-t}$**) (Noted: the needle-like microcrystals could be observed). (c) PXRD pattern of processed **Y$_{DMF-m}$/Y $_{DMF-t}$**.

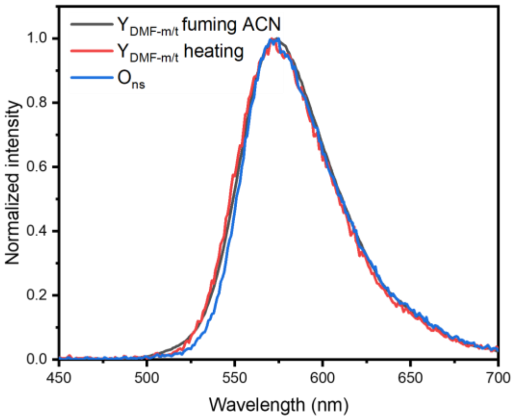

Figure S10. Emission spectra of **O$_{ns}$**, **Y $_{DMF-m}$/Y $_{DMF-t}$** heating and **Y $_{DMF-m}$/Y $_{DMF-t}$** fuming with ACN.

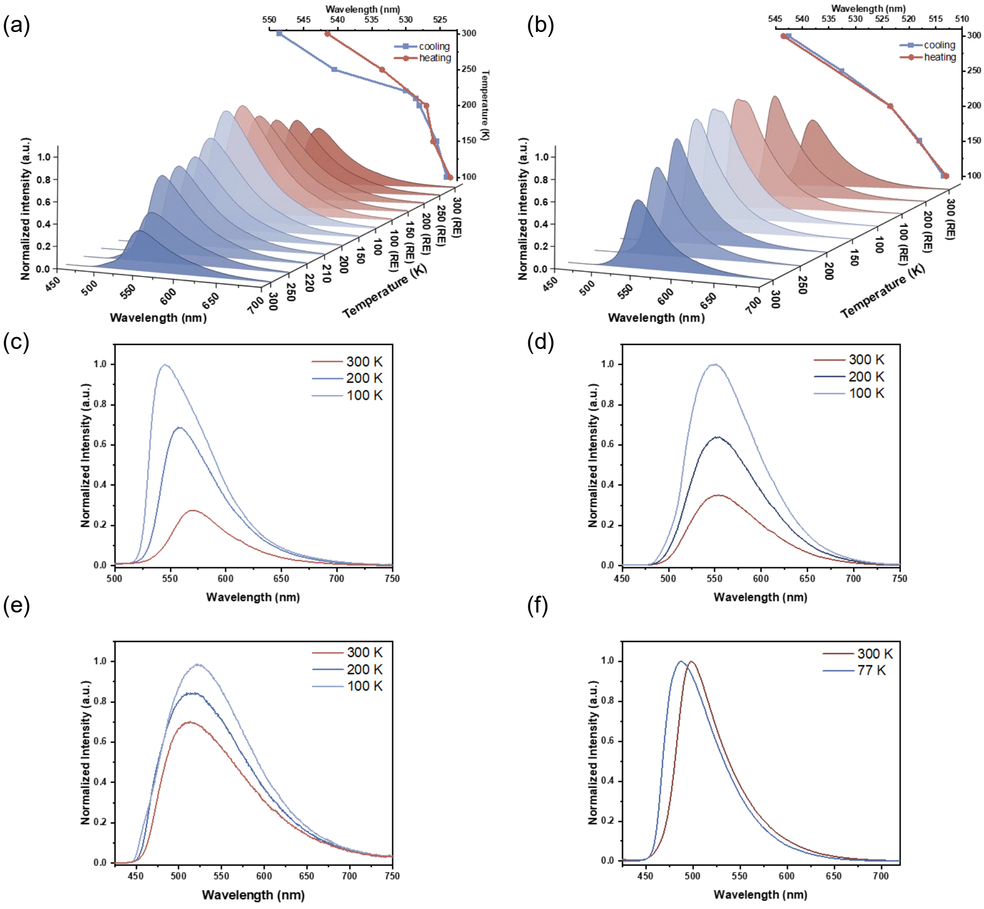

Figure S11. Temperature-dependent emission spectra of (a) **Y $_{DMF-m}$**; (b) **Y $_{DMF-t}$**; [Noted: "RE" means in rewarming process.] (c) **O$_{ns}$**; (d) **Y$_{ACN}$**; (e) **G$_{DMF}$**; (f) **G$_{ACN}$** (Normalized to 1).

Table S1. The photophysical data for crystals at 298 K

	λ_{em} ^{a} [nm] (Maximum emission wavlength)	Φ_{PL} ^{b} [%] (Quantitive yeild)
$\mathbf{O_{ns}}$	570	3.54
$\mathbf{Y_{ACN}}$	550	14.35
$\mathbf{Y_{DMF\text{-}m}}$	548	10.62
$\mathbf{Y_{DMF\text{-}t}}$	542	8.72
$\mathbf{G_{DMF}}$	510	23.20
$\mathbf{G_{ACN}}$	516	21.44

[a]λ_{ex} = 365 nm. [b]Measured using an integrating sphere method. (The absolute PL quantum efficiencies (Φ_{PL}) were determined using a Horiba FL-3018 Integrating Sphere.)

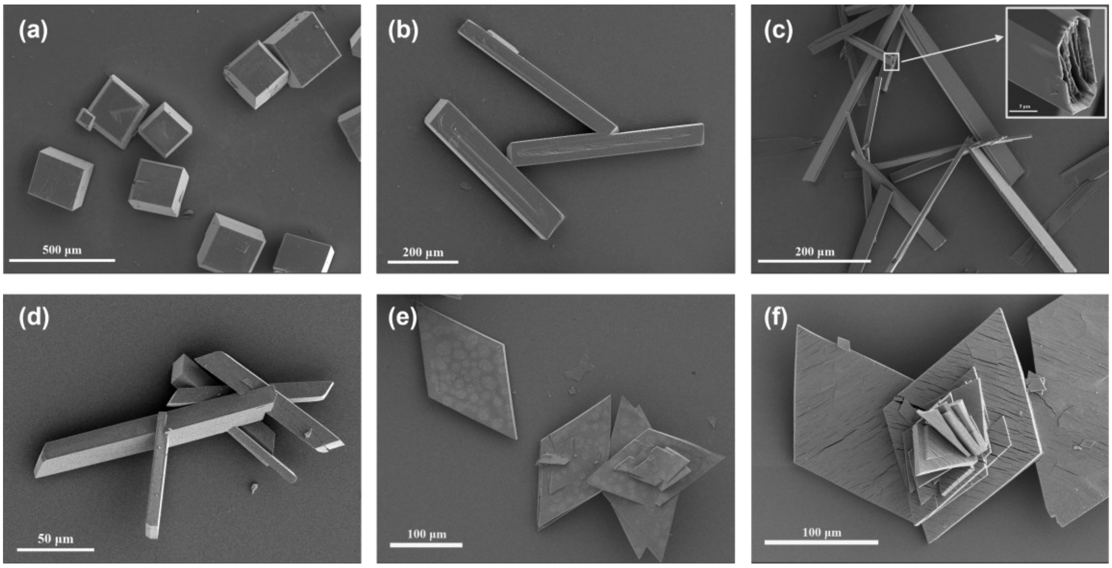

Figure S12. SEM photographs of (a) $\mathbf{Y_{DMF\text{-}m}}$; (b) $\mathbf{Y_{DMF\text{-}t}}$; (c) $\mathbf{O_{ns}}$; (d) $\mathbf{Y_{ACN}}$; (e) $\mathbf{G_{DMF}}$; (f) $\mathbf{G_{ACN}}$.

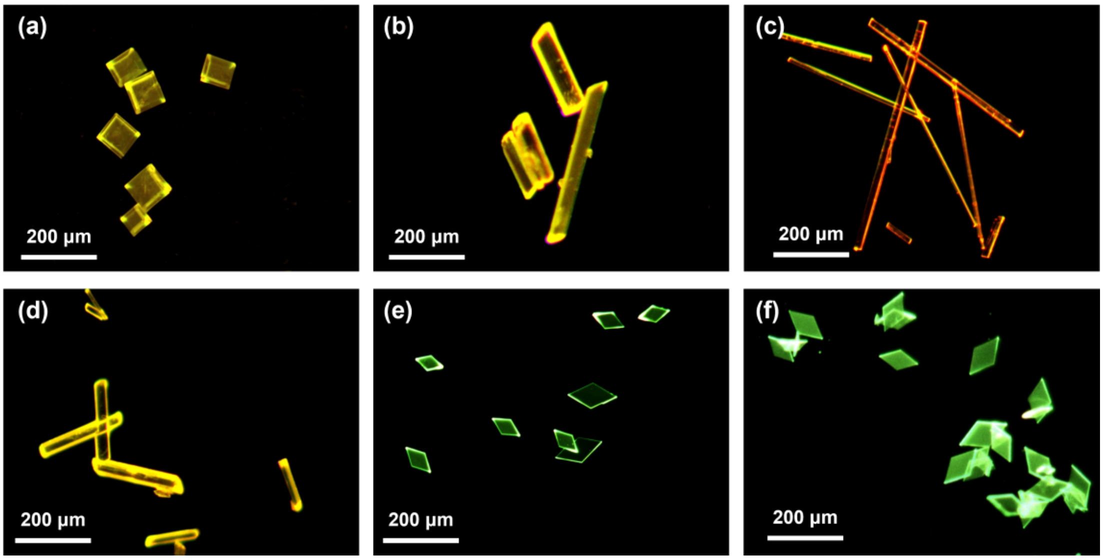

Figure S13. The luminescence microscope photographs of (a) **Y** $_{\mathbf{DMF\text{-}m}}$; (b) **Y** $_{\mathbf{DMF\text{-}t}}$; (c) **O** $_{\mathbf{ns}}$; (d) **Y** $_{\mathbf{ACN}}$; (e) **G** $_{\mathbf{DMF}}$; (f) **G** $_{\mathbf{ACN}}$ were taken under UV irradiation (365 nm).

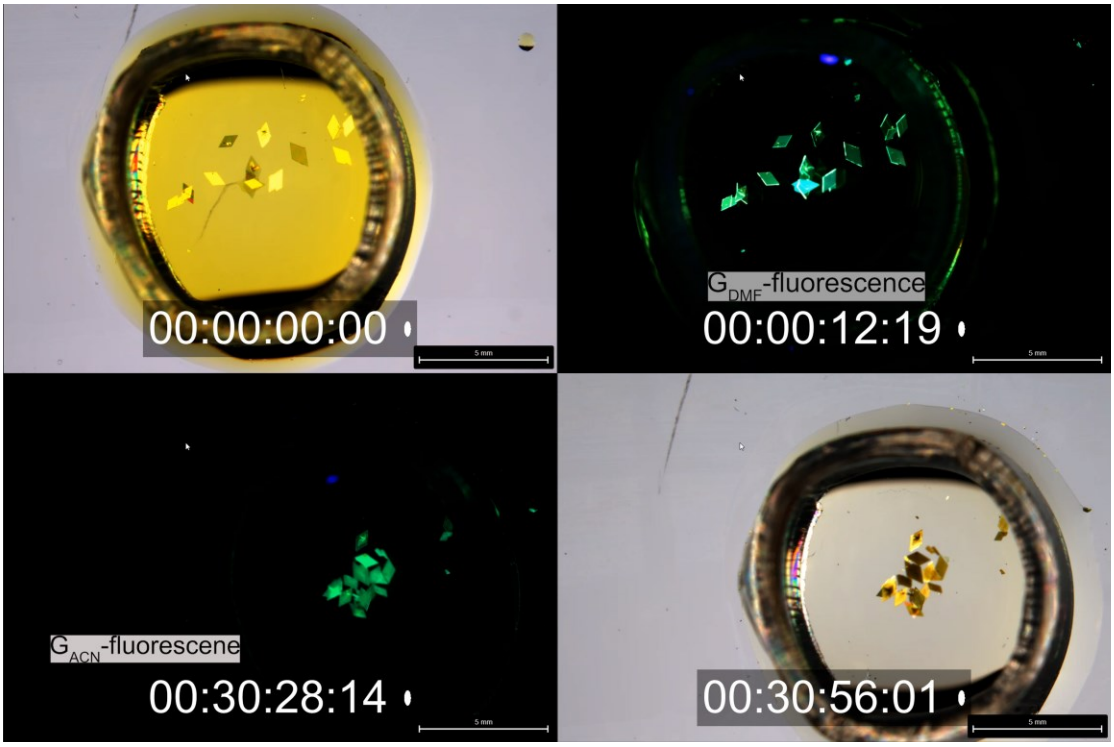

Figure S14. The screenshots from the Video. Note: Add some **G** $_{\mathbf{DMF}}$ with DMF solution into the sample cell (to keep the crystals from floating away), then remove the mother liquid and add ACN pure solvent. Due to its poor solvability in ACN, the crystals would not dissolve. After 30 min, they transformed into **G** $_{\mathbf{ACN}}$, retaining the original shapes and similar fluorescence.

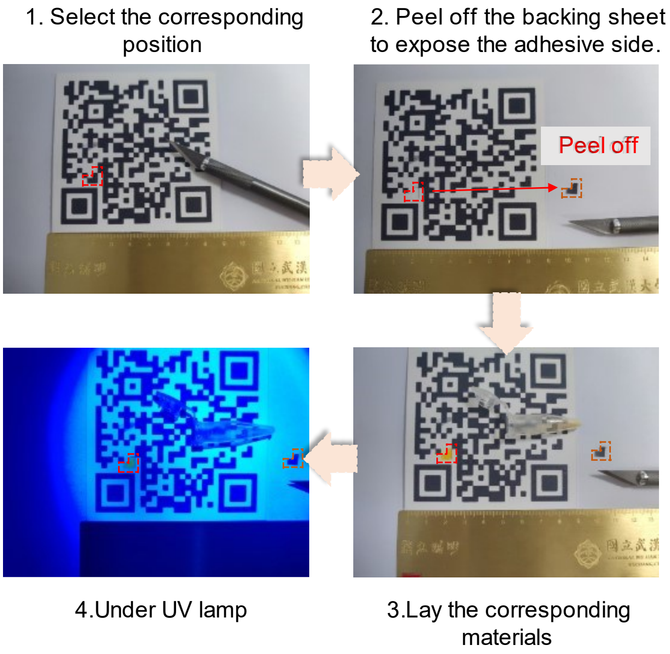

Figure S15. The steps to prepare the QR code paper.

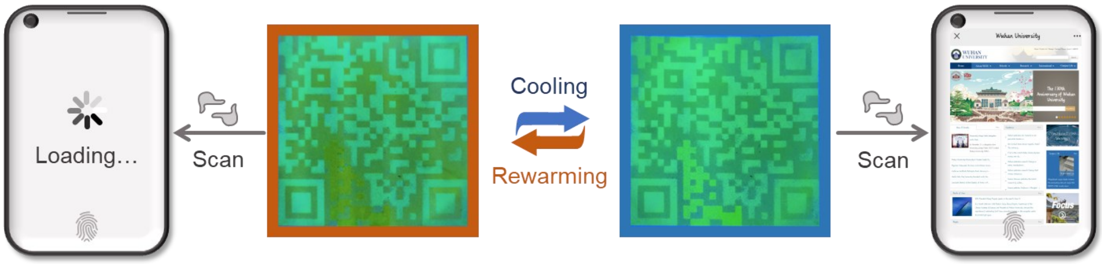

Figure S16. The temperature-dependent image encryption. (The photographs in the middle were taken with a smartphone, under UV light (365 nm).)

III. Crystal structures analysis.

Scheme S2. Three types of phenyl ring packing mode.

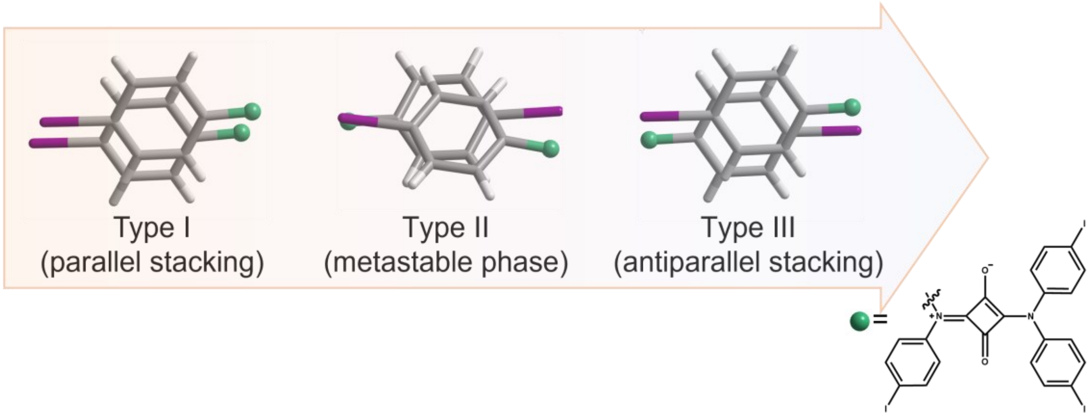

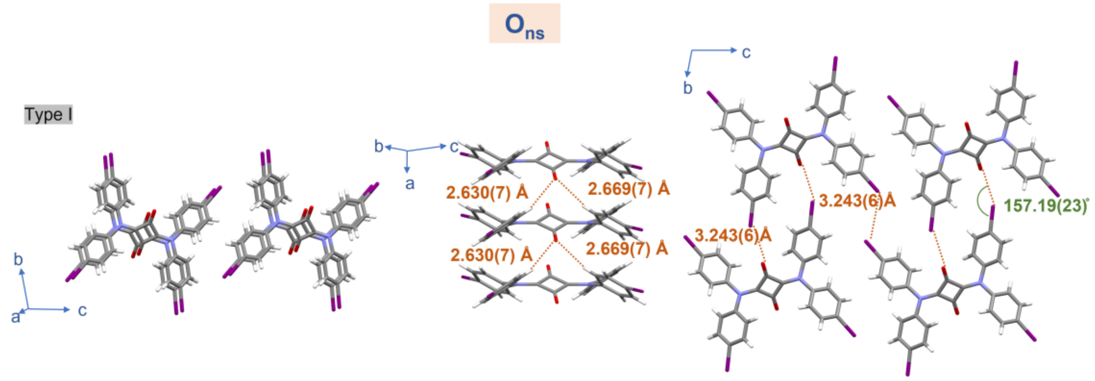

Figure S17. The $\mathbf{O_{ns}}$ belonged to triclinic crystal system and crystallized in *P*−1 space group taking the parameters of a= 4.2913(2) Å, b= 12.0541(5) Å, c= 13.8643(8) Å. It was noteworthy that the SQD-I stacked in a parallel mode-type Ⅰ and the paired XBs [3.243(6) Å, 157.19(23)°] between two SQD-I played a significant role to form the linear arrangement and the two hydrogen bonds [2.669(7) Å and 2.630(7) Å] were the major reason to pack in completely "face to face" mode. It should be pointed out that the distance between the two free iodine is 4.685(1) Å, which allows the structure respond to solvent stimulation.

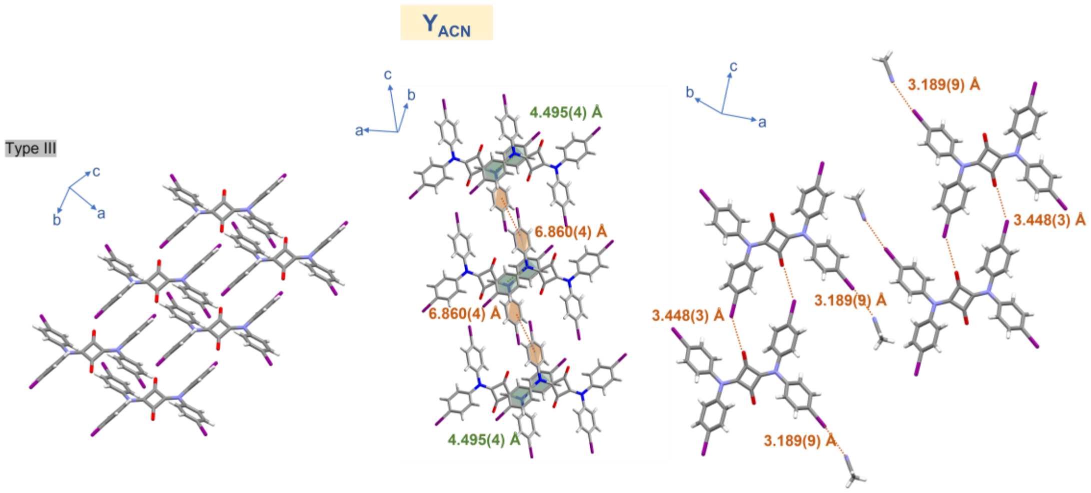

Figure S18. The $\mathbf{Y_{ACN}}$ belonged to triclinic crystal system and crystallized in *P*-1 space group taking the parameters of a= 7.3219(4) Å, b= 9.9426(6) Å, c= 12.1517(6) Å. All four iodine atoms have formed halogen bonds, and two of them are between SQD-I [3.448(3) Å], the other two are with ACN [3.189(9) Å].

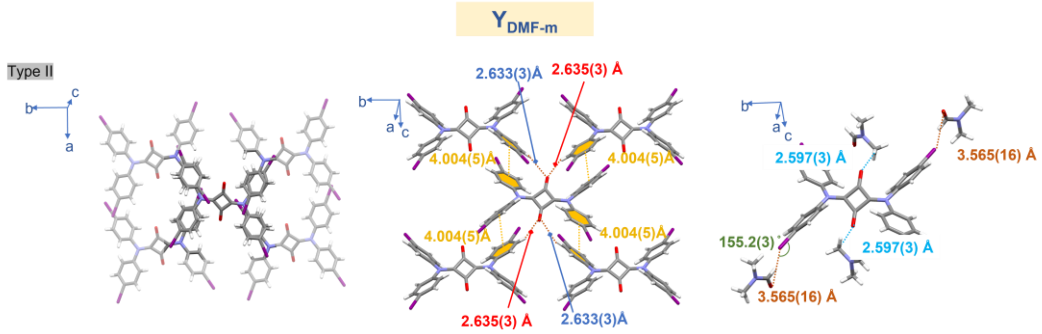

Figure S19. The $\mathbf{Y_{DMF\text{-}m}}$ crystal belonged to monoclinic crystal system and crystallized in *P*2$_1$/*n* space group taking the parameters of a= 11.0008(4) Å, b= 14.4283(5) Å, c= 12.4684(5) Å. The SQD-I stacked in a metastable phase which means not completely antiparallel stacking. The π-π interaction [4.051(3) Å] and two hydrogen bonds [2.633(3) Å, 2.635(3) Å] are formed to lock the phenyl rings rotation. From the value of interactions, the four phenyl rings are in a relatively uniform. The distance [3.565(16) Å] and angle [155.2(3)º] fall outside the typical range defined for halogen bond. The H of methyl group formed hydrogen bond [2.597(3) Å] with oxygen sites.

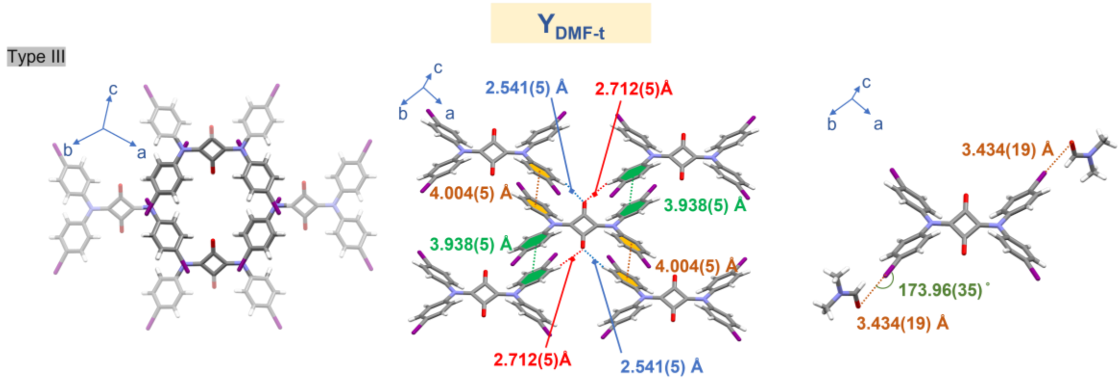

Figure S20. The $\mathbf{Y_{DMF\text{-}t}}$ crystal belonged to triclinic crystal system and crystallized in *P*−1 space group taking the parameters of a= 9.9675(4) Å, b= 10.1596(4) Å, c= 10.7649(4) Å. The stacking mode is completely antiparallel-type III. The π-π interaction [4.004(5) Å, 3.938(5) Å] and two hydrogen bonds [2.541(5) Å, 2.712(5) Å] became inequivalent compared to $\mathbf{Y_{DMF\text{-}m}}$ [2.633(3) Å, 2.635(3) Å]. And the two of iodo-groups formed halogen bonds [3.434(19) Å, 173.96(35)°].

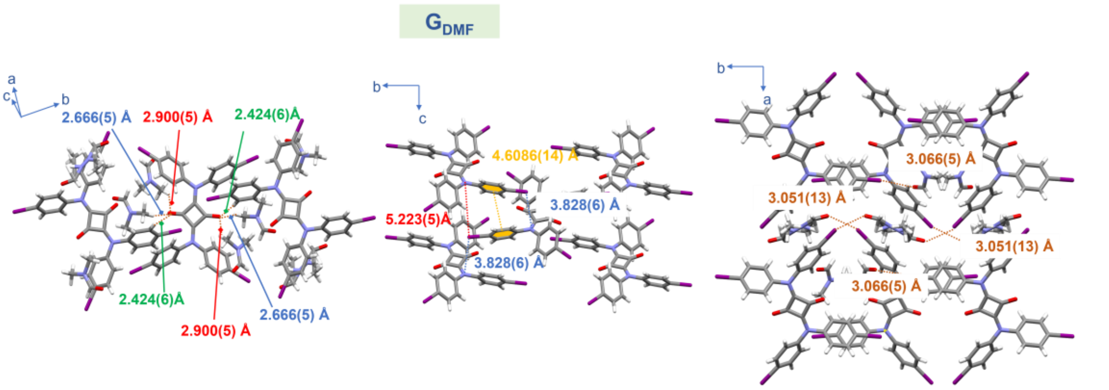

Figure S21. The **G$_{DMF}$** crystal belonged to monoclinic crystal system and crystallized in *P*2$_1$/*c* space group taking the parameters of a= 13.9680(15) Å, b= 16.7251(11) Å, c= 8.9232(8) Å. The halogen bonds between O(DMF) and I(SQD-I) are significantly shorter than those observed in other crystals. Additionally, one of the DMF molecules undergoes back-and-forth movement, resulting in the formation of halogen bonds between only three iodo-groups of SQD-I in average. The packing of the two phenyl rings is not strictly parallel stacked.

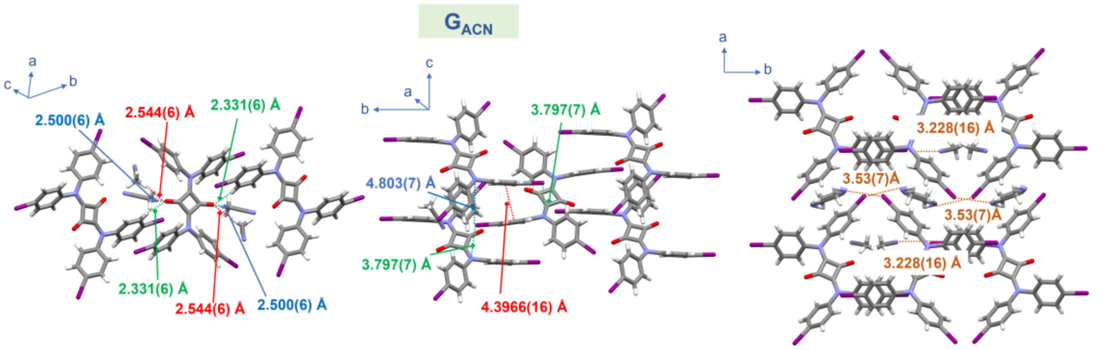

Figure S22. The **G$_{ACN}$** crystal belonged to monoclinic crystal system and crystallized in *P*2$_1$/*c* space group taking the parameters of a= 13.8266(17) Å, b= 16.4286(12) Å, c= 8.5555(10) Å. The halogen bonds between N(ACN) and I(SQD-I) are 3.53(7) Å and 3.228(16) Å. Additionally, the packing of the two phenyl rings is closer and more parallel than in **G$_{DMF}$**.

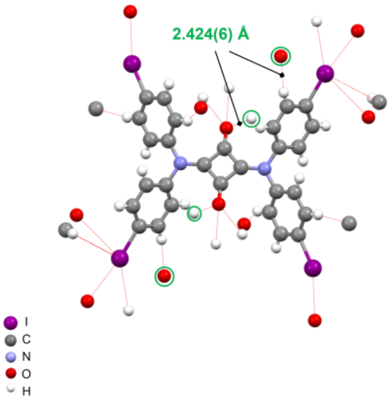

Figure S23. In G_{DMF}-200 K**:** The weak interactions involved in a SQD-I molecule (shorten than the sum of vdW radii, identified with Mercury 2023. 3. 0). The pairs of 2.424(6) Å HB are connected to the other SQD-I molecules via the atoms with green circles labeled. The other weak bonds are all contacted with solvent molecules (DMF).

Table S2. The torsional angle (θ_1, θ_2, θ_3 and θ_4) and the pitch angle (α).

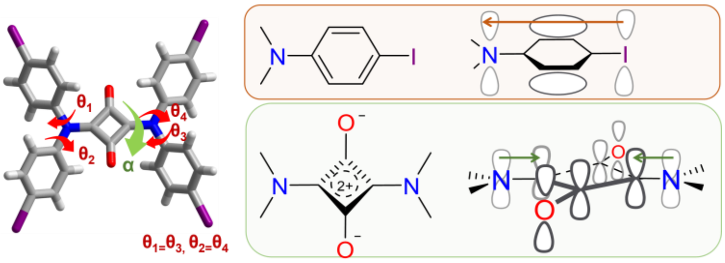

Y_{DMF-m}	θ_1	θ_2	α
300 K	34.23	41.43	22.13
250 K	34.32	41.05	21.91
200 K	37.37	38.16	22.27
100 K	37.62	37.21	22.15
200 K-RE	38.07	37.48	22.37
300 K-RE	38.72	36.26	22.49

	θ_1	θ_2	α
O_{ns}-300 K	35.21	35.24	28.30
Y_{ACN}-300 K	40.79	44.07	19.25
G_{DMF}-200 K	43.23	51.44	13.36
G_{ACN}-200 K	40.42	52.96	12.81

Y_{DMF-t}	θ_1	θ_2	α
300 K	38.01	37.43	22.18
250 K	38.01	37.92	22.37
200 K	37.13	37.42	22.22
150 K	38.77	36.50	21.76
100 K	37.78	37.74	21.65
150 K-RE	37.39	37.47	22.07
200 K-RE	37.42	37.45	22.39
250 K-RE	38.50	37.82	21.82
300 K-RE	39.27	36.86	22.24

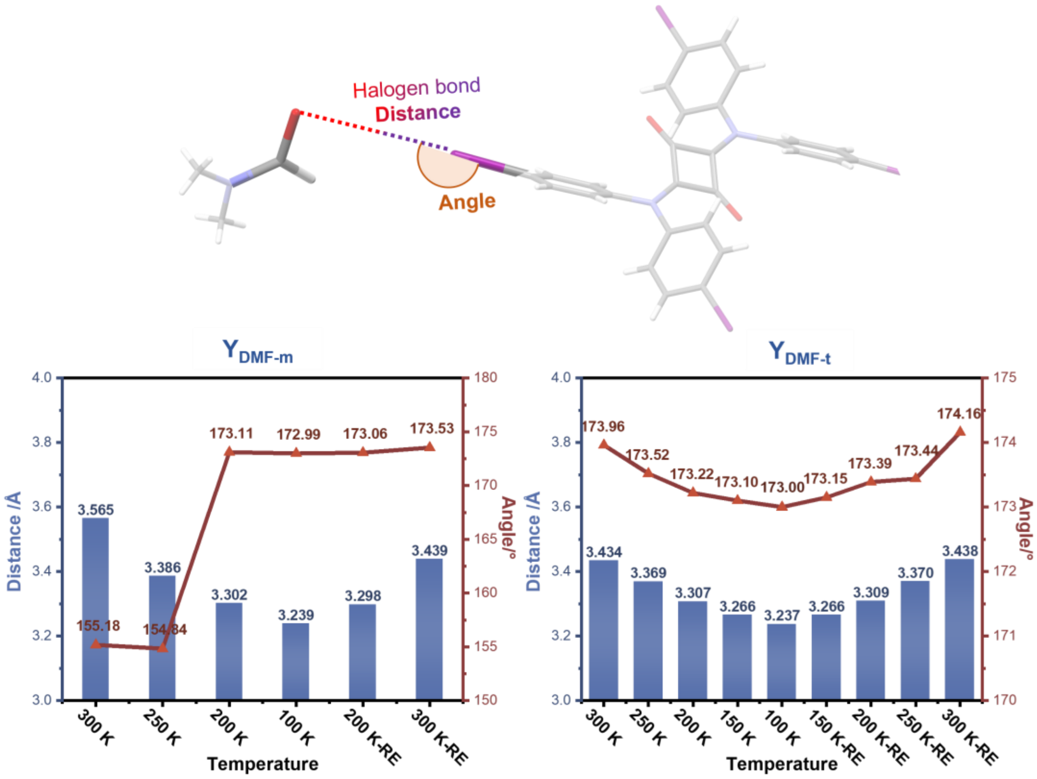

Figure S24. The temperature-dependent halogen bond distance and angle in **Y** $_{\mathbf{DMF\text{-}m}}$ and **Y** $_{\mathbf{DMF\text{-}t}}$.

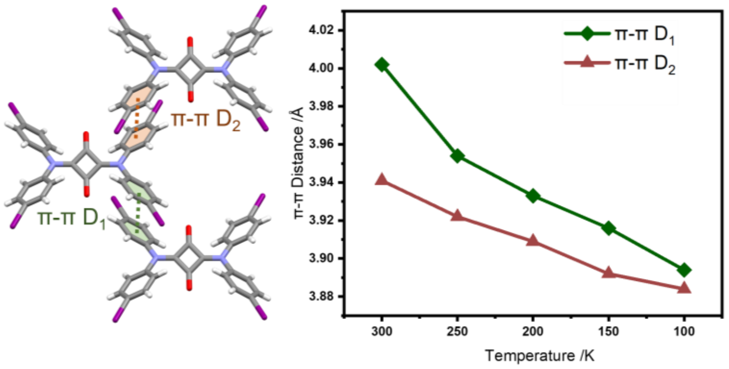

Figure S25. The distance between two centroids of phenyl groups in **Y** $_{\mathbf{DMF\text{-}t}}$ at different temperatures.

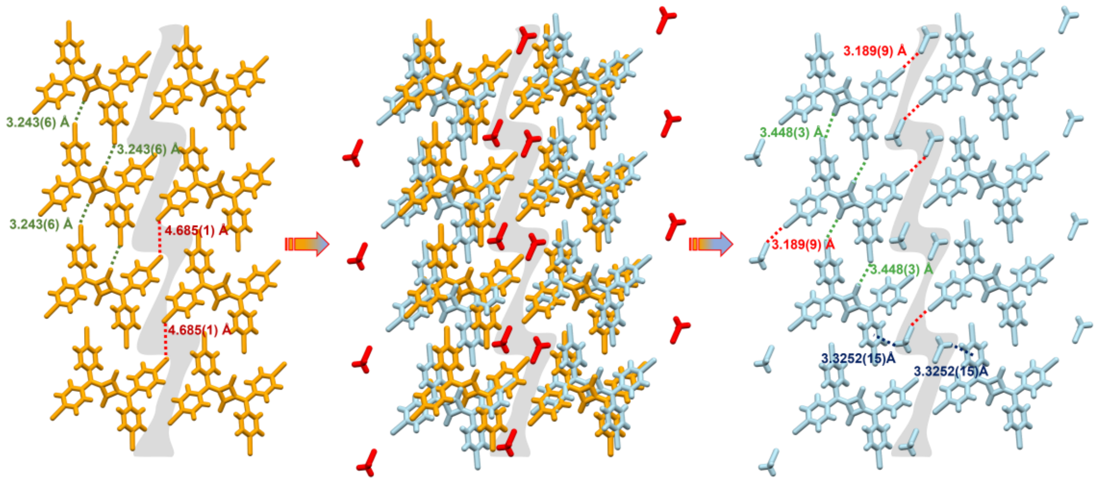

Figure S26. The crystal recrystallization from $\mathbf{O_{ns}}$ to $\mathbf{Y_{ACN}}$. (orange=$\mathbf{O_{ns}}$, blue=$\mathbf{Y_{ACN}}$, the red ACN molecules simulate the process of ACN solvent entering gray channels.)

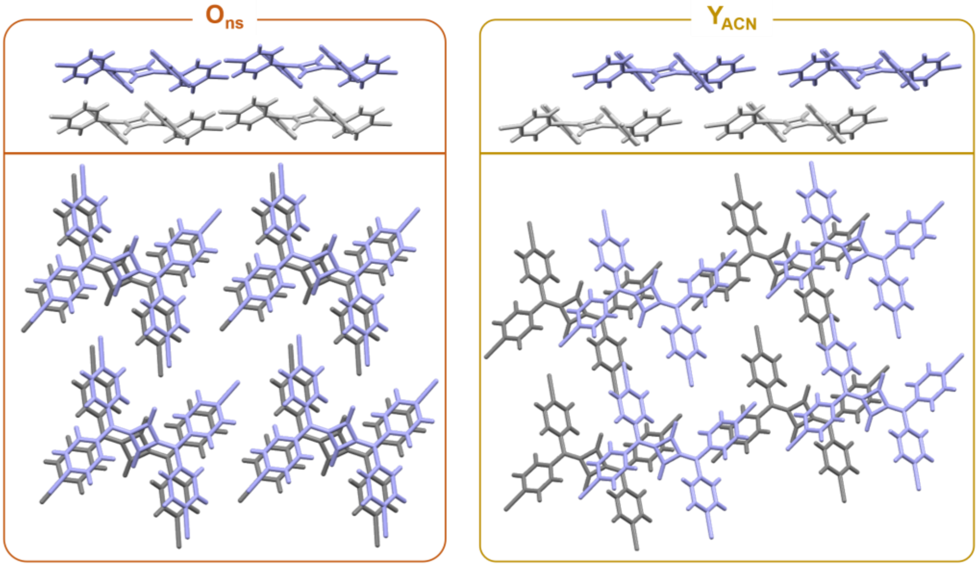

Figure S27. The slipping process from $\mathbf{O_{ns}}$ to $\mathbf{Y_{ACN}}$.

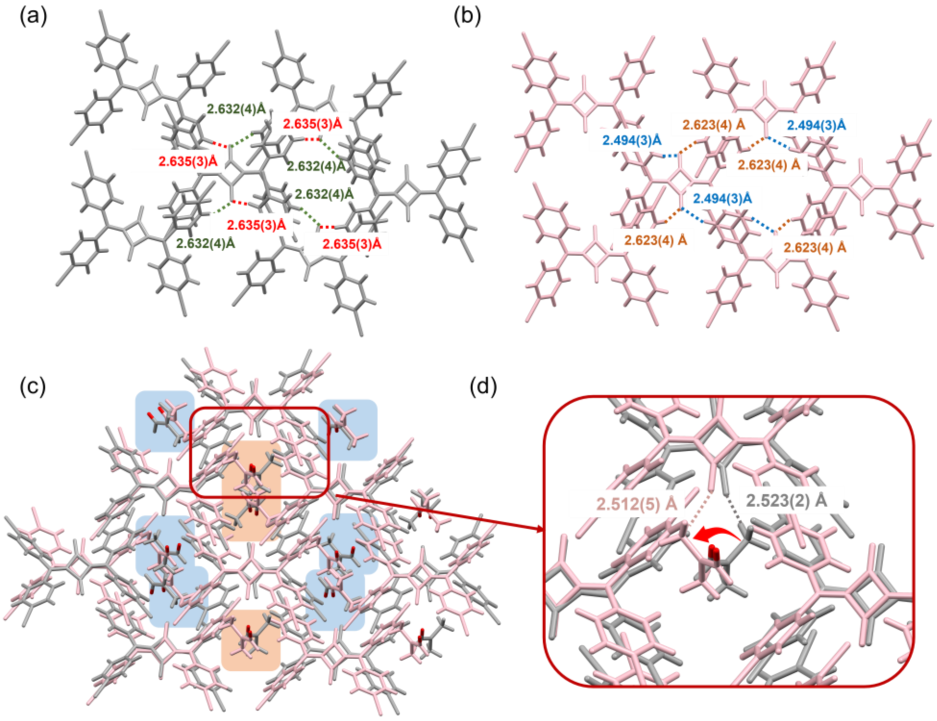

Figure S28. The SCSC transformation from $\mathbf{Y_{DMF\text{-}m}}$**-250 K** (monoclinic) to $\mathbf{Y_{DMF\text{-}m}}$**-200 K** (triclinic). (gray= $\mathbf{Y_{DMF\text{-}m}}$**-250 K**, pink= $\mathbf{Y_{DMF\text{-}m}}$**-200 K**). (a) and (b) the hydrogen bond of $\mathbf{Y_{DMF\text{-}m}}$**-250 K** and $\mathbf{Y_{DMF\text{-}m}}$**-200 K**. (c) The change of DMF alignment (The DMF with orange background turned around, the blue background kept the same direction.) (d) The hydrogen bond distance of (DMF)CH_3 ···· O(SQD-I).

After rewarming to 300 K, the persistence of the triclinic space group indicates that $\mathbf{Y_{DMF\text{-}t}}$ is thermodynamically more stable than $\mathbf{Y_{DMF\text{-}m}}$. Therefore, $\mathbf{Y_{DMF\text{-}m}}$ is more likely a kinetically favored arrangement, supported by the observation that ultrasonication could reduce the conversion time from $\mathbf{O_{ns}}$ powder to $\mathbf{Y_{DMF\text{-}m}}$ microcrystals (less than 30 seconds).

III. XANES (X-ray absorption near edge structure) experiment

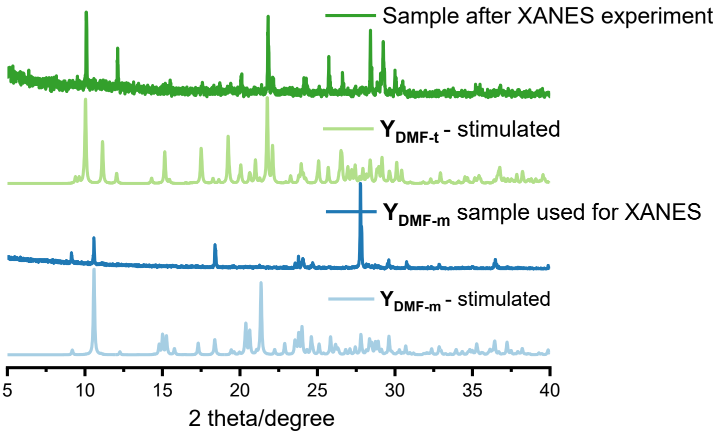

Figure S29. The PXRD patterns of samples used for the XANES experiment.

XANES

1. Experimental Setup

The XANES measurements were performed at XAFCA beamline of Singapore Synchrotron Light Source where the storage ring is running at 0.7 GeV with current 150 mA.[20] The sample was placed in the X-ray beam path, and care was taken to minimize any potential sources of interference.

2. Energy Calibration

Before starting data collection, we calibrated the X-ray energy using a titanium foil, aligning it with the Titanium K edge XANES energy of 4996 eV. This calibration is notably close to the L3 edge XANES energy of Iodine, which is approximately 4557 eV.

3. Data Collection:

The measurements were carried out in fluorescence mode. The fluorescence yields from the sample were measured using a silicon drift detector, Bruker XFlash6|100.

4. Temperature Control:

The temperature-dependent studies were conducted, the sample chamber has been equipped with a temperature control system, LakeShore 336 Temperature Controller, to vary the temperature during measurements.

5. Data Analysis:

The program Athena of the Demeter software package[21] was used to align and normalize XANES data.

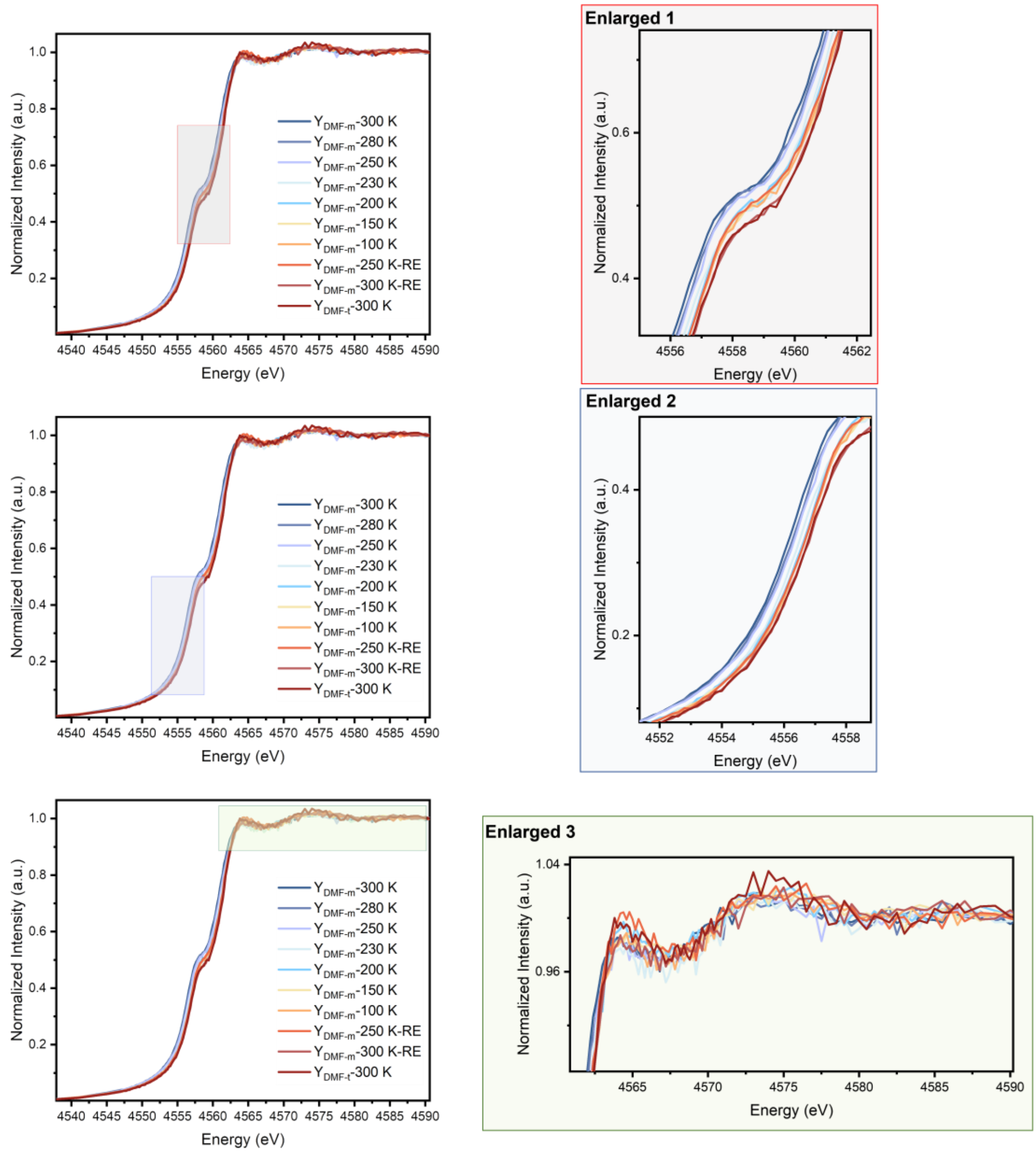

Figure S30. The Normalized XANES spectra and inset. (We used **Y_{DMF-m}** at beginning, then cooling to 100 K, and rewarming back to 300 K, the data collected during the rewarming process is referred to as 'RE'. Finally, we use other Y_{DMF-t} sample to measure at 300 K, and it almost consisted with **Y_{DMF-m}**-300 K-RE (which has transformed into triclinic crystal system already).

IV. Calculations.

i. RDG Analysis

For RDG analysis the geometry of dimers taken from the crystal structures was used and optimization of the hydrogen atoms was performed at DFT (ωB97X-D3/def2TZVP)[22,23] level using *ORCA* 5.0.3.[24] Based on the resulting wavefunction, the RDG analysis[25] was performed using *MultiWFN*[26]. A grid box representing the region of interaction was chosen with a grid spacing of 0.1 Bohr. Graphical representations were rendered using *VMD* 1.9.3.[27] (isovalue = 0.5) and scatter plots were generated using *gnuplot*. The Reduced Density Gradient (RDG) analysis, as presented in Figures S31-S33, was employed to investigate the non-covalent interactions within the crystal structure of **O$_{ns}$**-300 K. RDG isosurfaces and corresponding scatter graphs were rendered to visualize these interactions. RDG analysis is particularly useful for identifying and characterizing weak interactions like hydrogen bonds and van der Waals forces. The isosurfaces and scatter plots illustrate the nature and strength of these interactions within the crystal structure.

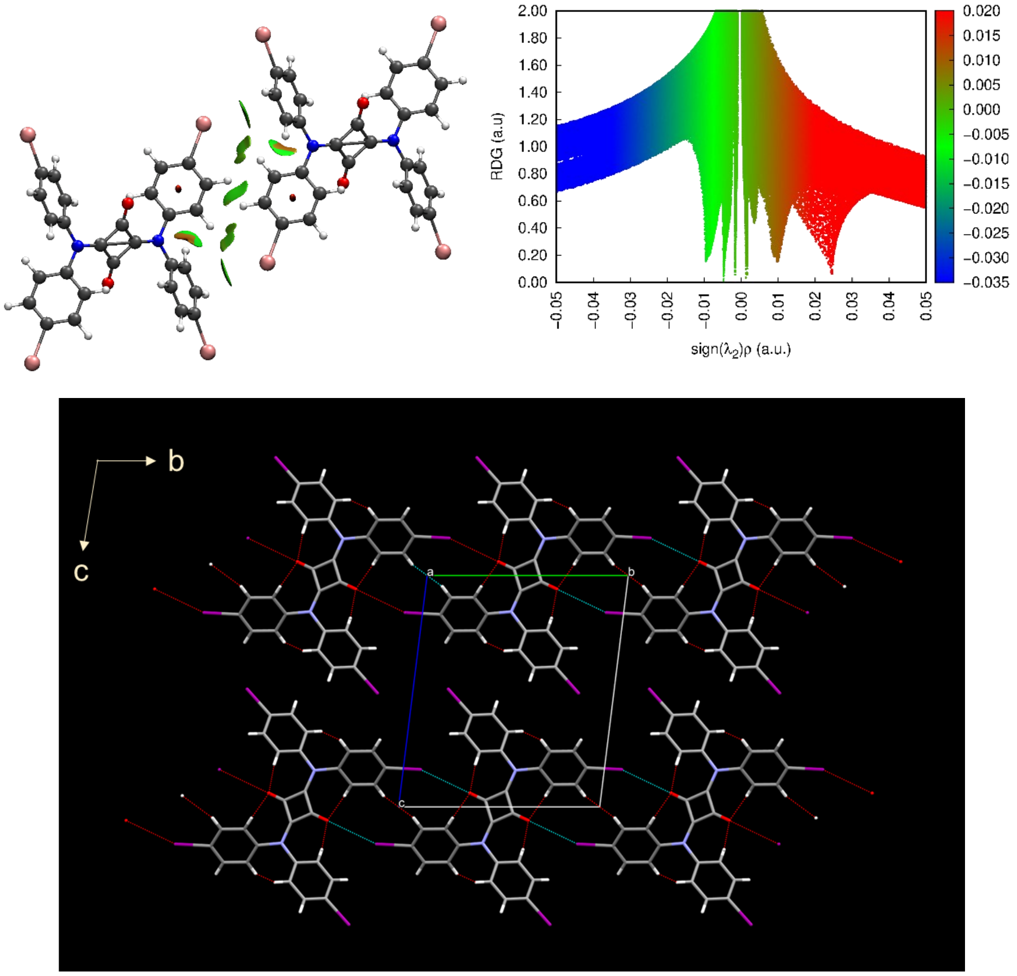

Figure S31. Rendering of the RDG isosurfaces between a dimer in the crystal structure of **O$_{ns}$**-300 K (left). Plot of the corresponding RDG scatter graph (right). Crystal analysis in *Mercury* (bottom).

These isosurfaces imply the presence of weak interactions between the two SQD-I molecules along axis c, although these interactions cannot be identified in Mercury

2022.3.0 by being shorter than the van der Waals radius.

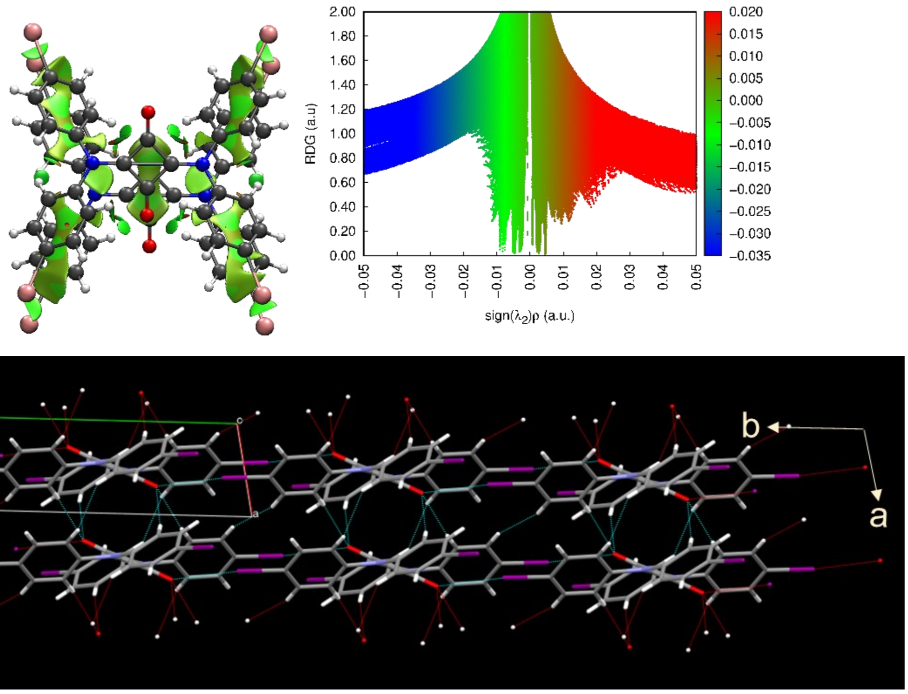

Figure S32. Rendering of the RDG isosurfaces between a dimer in the crystal structure of **O**$_{ns}$-300 K (left). Plot of the corresponding RDG scatter graph (right). Crystal analysis in *Mercury* (bottom).

The non-covalent bond interactions were pp interactions and HBs along axis a.

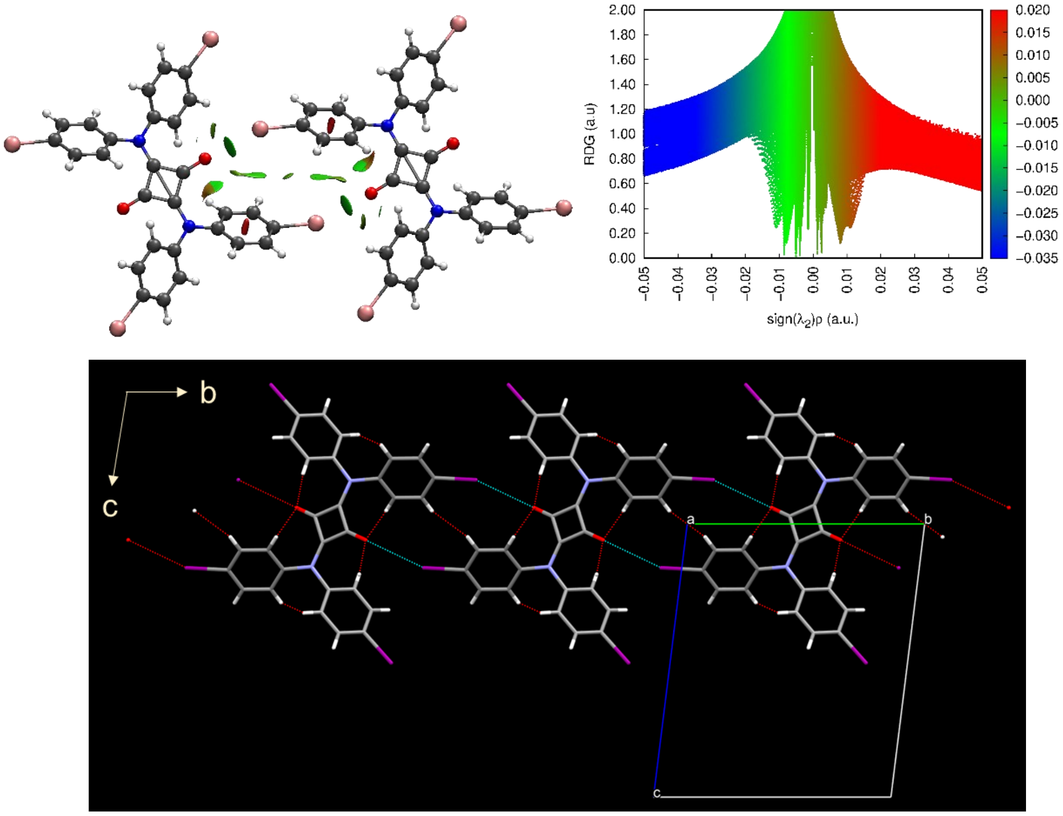

Figure S33. Rendering of the RDG isosurfaces between a dimer in the crystal structure

of **O_{ns}**-300 K (left). Plot of the corresponding RDG scatter graph (right). Crystal analysis in *Mercury* (bottom).

The non-covalent bond interactions were XBs between I and O along axis b.

ii. Hirshfeld Surface Analysis.

Hirshfeld Surface Analysis[13] provides insights into the multiple-dimensional intermolecular interactions in crystals, which is crucial for understanding the molecular packing and its influence on the properties of the material. Here we performed on CrystalExplorer.[28] To obtain more insight of the multiple-dimensional intermolecular interactions, two-dimensional (2D)-fingerprints of crystals and the associated Hirshfeld surfaces were employed to show their intermolecular interactions. The red and blue regions on the Hirshfeld surfaces represent high and low close contact populations, respectively. Distances to the nearest atoms outside (intermolecular, d_e) and inside (intramolecular, d_i) are used for color-coded mapping of the surface. For each point on the Hirshfeld surface, the normalized contact distance (d_{norm}) was determined by ***eq 1*** in which d_i and d_e are measured from the surface to the nearest atom interior and nearest atom exterior to the surface interior, respectively, where r_i^{vdW} and r_e^{vdW} are the van der Waals radii of the atoms. That is to say, the value of d_{norm} is negative or positive depending on the intermolecular contacts being shorter or longer than the van der Waals separations. Therefore, through the location of ($d_i d_e$) points and their relative frequencies discernible on the surface and the 2D fingerprint plot, it is possible to ascertain the distances and intensities of intermolecular interactions.

$$d_{norm} = (d_i - d_i^{vdW})/r_i^{vdW} + (d_e - d_e^{vdW})/r_e^{vdW} \qquad \textbf{\textit{(1)}}$$

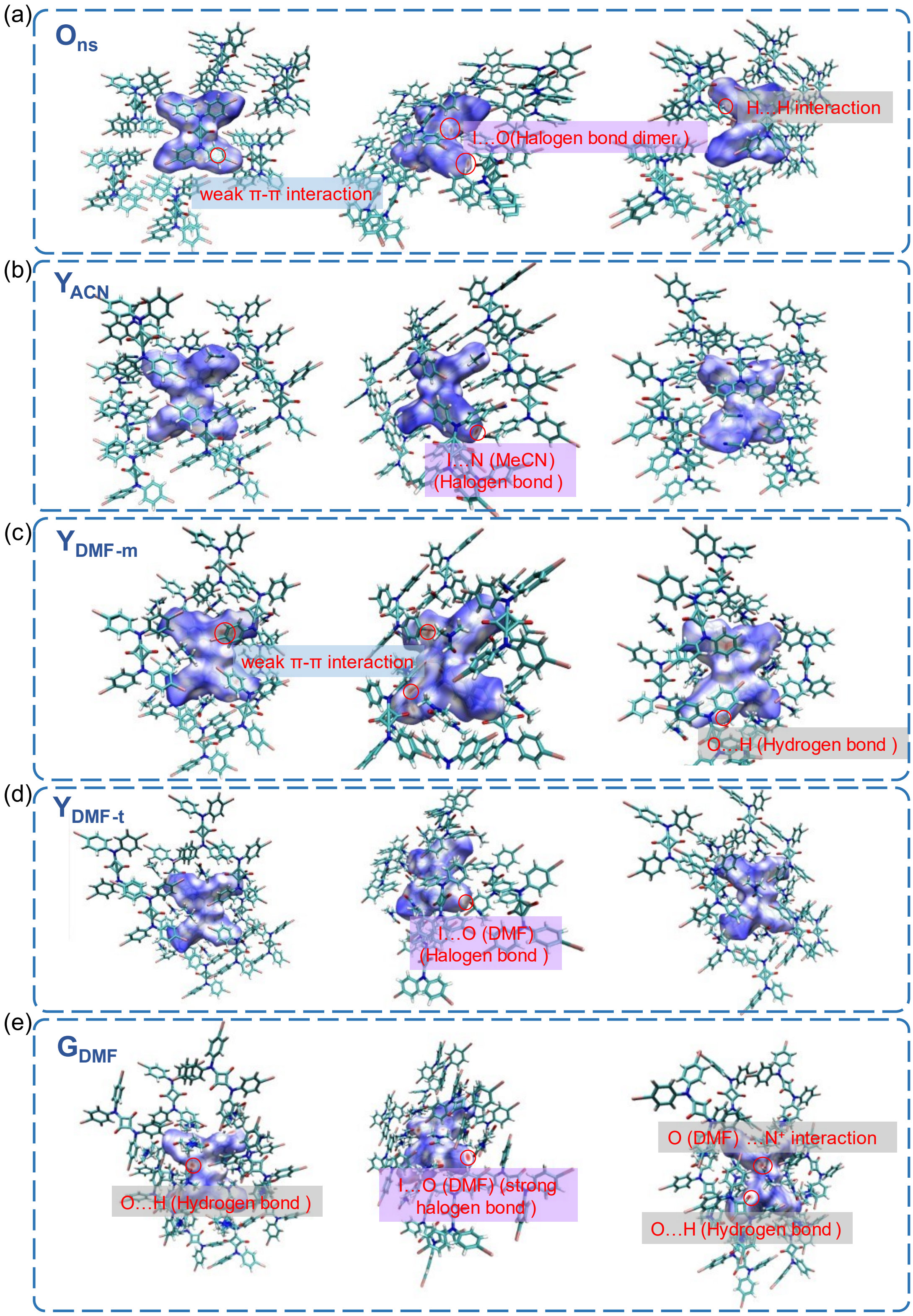

Figure S34. The Hirshfeld Surface Analysis of SQD-I in crystals of (a) $\mathbf{O_{ns}}$; (b) $\mathbf{Y_{ACN}}$; (c) $\mathbf{Y_{DMF\text{-}m}}$; (d) $\mathbf{Y_{DMF\text{-}t}}$ and (e) $\mathbf{G_{DMF}}$.

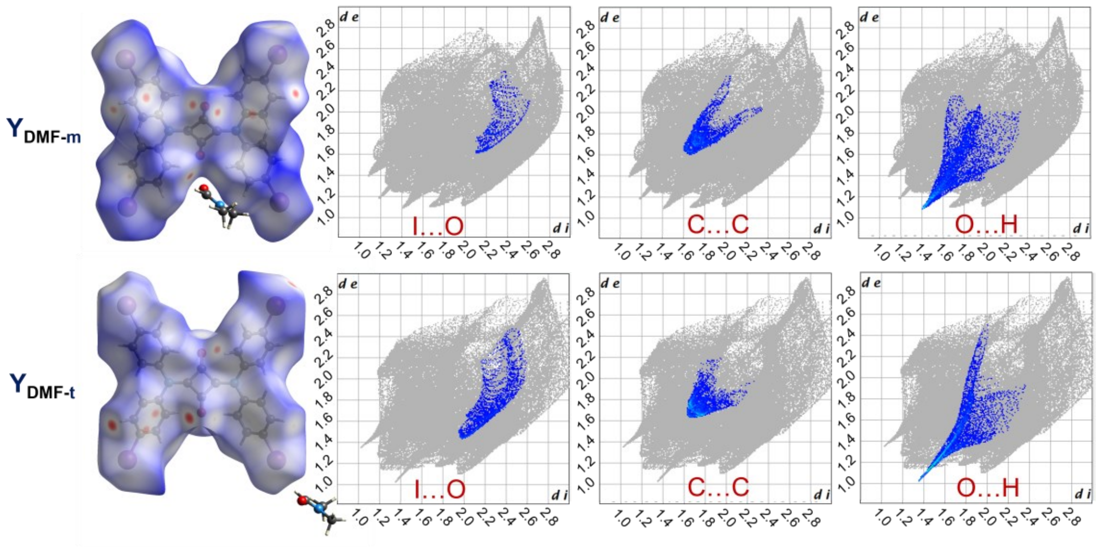

Figure S35. Hirshfeld surface and 2D fingerprint plots of $\mathbf{Y_{DMF\text{-}m}}$ and $\mathbf{Y_{DMF\text{-}t}}$.

iii. Frontier orbitals calculations

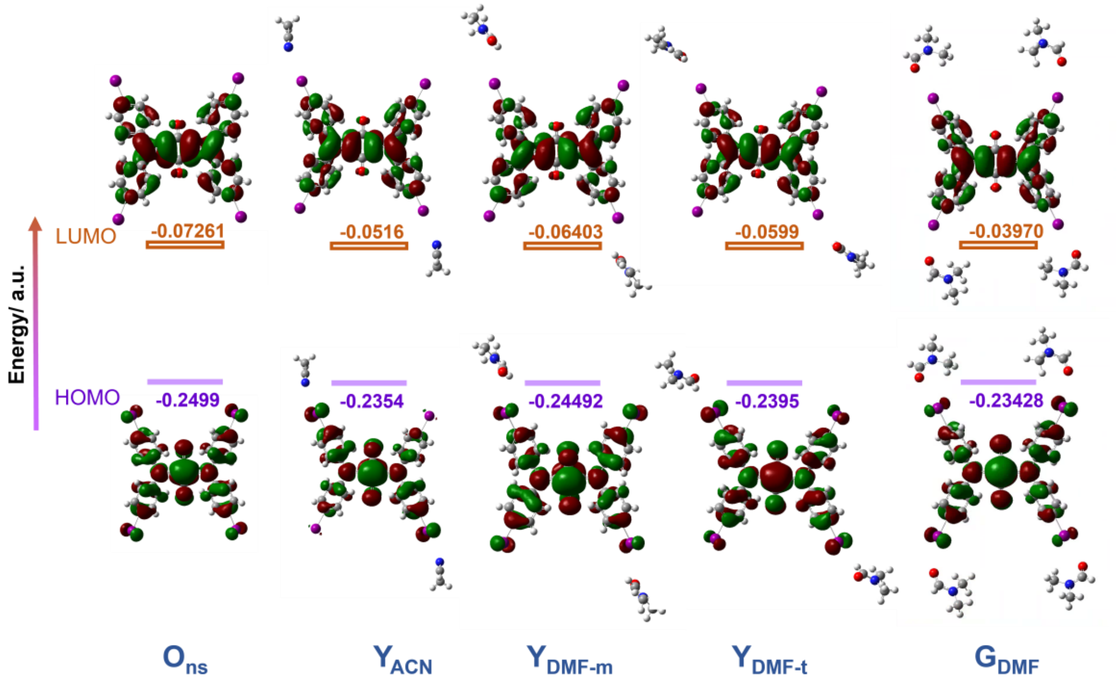

Figure S36. Schematic energy diagrams and frontier molecular orbitals for $\mathbf{O_{ns}}$, $\mathbf{Y_{ACN}}$, $\mathbf{Y_{DMF\text{-}m}}$, $\mathbf{Y_{DMF\text{-}t}}$ and $\mathbf{G_{DMF}}$. (They were performed using Gaussian software.[29] The density functional theory (DFT) method was employed with the B3LYP functional, accompanied by the def2-TZVP basis set. The B3LYP functional includes both Hartree-Fock exchange and gradient-corrected correlation. The def2-TZVP basis set further enhances the quality of the results by providing a comprehensive description of valence electrons with the inclusion of polarization functions.)

The calculations for the rotation energy profile were conducted using Gaussian software, utilizing the B3LYP functional with the def2-TZVP basis set. This choice of method and basis set was intended to balance computational efficiency with the accuracy required for our system of interest. In our analysis, we employed a relaxed scan approach for the dihedral angles. During this process, the dihedral angle under investigation was incrementally varied, and at each step, all other geometrical parameters of the molecule were allowed to optimize. This approach was chosen to more accurately reflect the potential energy surface of the molecule as it would exist in a realistic environment, allowing for the natural relaxation of the molecular structure in response to the changes in the dihedral angle. The optimization at each step ensures that we are locating the nearest local minimum, which provides a true representation of the energy barrier associated with the rotation. The Gaussian input file contained the 'Opt=ModRedundant' directive, which facilitated the relaxation of all other geometrical parameters except for the specified dihedral angle.

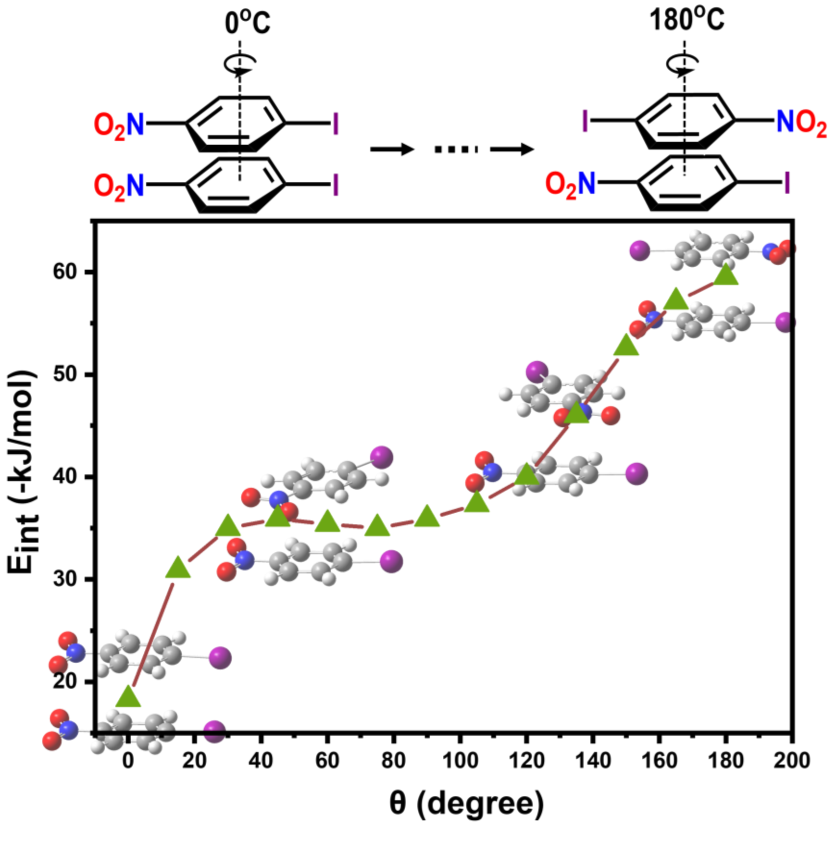

Figure S37. Rotation angle versus interaction energy for 4-nitroiodobenzene molecules. Note: For simplicity, the 4-iodo-aminophenyl group has been replaced with 4-nitroiodobenzene. The plot illustrates the shift in interaction energy between a pair of 4-nitroiodobenzene molecules as the stacking angle varies. A zero-degree angle denotes a parallel arrangement. Upon rotating the molecules incrementally towards a 180-degree alignment, a notable escalation in interaction energy is observed. This provides a clear understanding of the angular dependence of interaction energy in this molecular system.

iv. Electrostatic potential (ESP) Analysis.

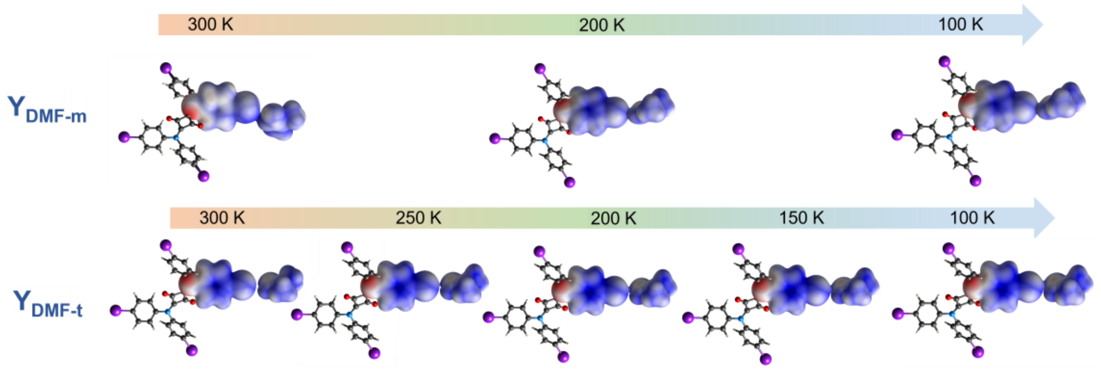

Figure S38. Conformations and corresponding V_S representations of electron density isosurfaces for temperature-dependent SQD-I and DMF in $\mathbf{Y_{DMF-m}}$ or $\mathbf{Y_{DMF-t}}$ employed CE-B3LYP/def2-TZVP.[23] (In these V_S representations, color variations indicate differences in V_S values: shades leaning towards red represent positive V_S, shades leaning towards blue signify negative V_S, and intermediate V_S values are shown in white. These visualizations provide valuable insights into the spatial arrangement and electronic properties of the system under various temperature conditions.)

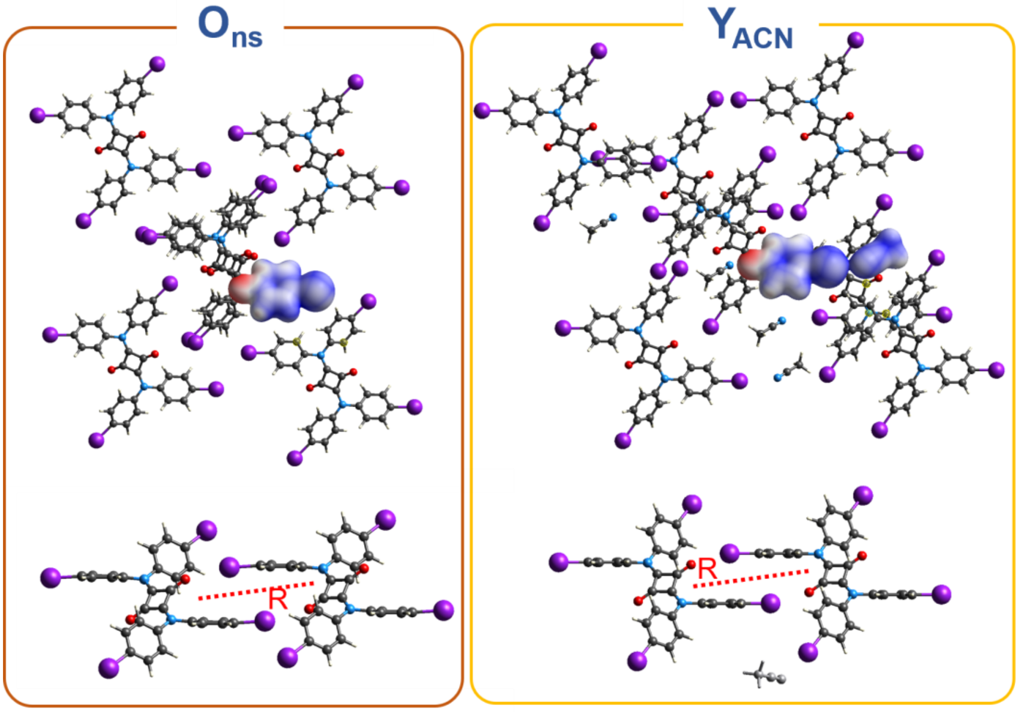

	R	E_{ele}	E_{pol}	E_{int}
O_{ns}	12.05	5.9	-5.2	-37.6
Y_{ACN}	12.15	1.7	-4.3	-39.8

Figure S39. Employed CE-B3LYP/def2-TZVP (CrystalExplorer).[23] Interaction Energies (kJ/mol): The following energy components are detailed: E_{ele}- Electrostatic

Energy, E_{pol}- Polarization Energy, and E_{int}- Interaction Energy. 'R' indicates the distance between the centers of mass of the molecules, measured in Angstroms (Å). These measurements help delineate the relationships between these energy components and their influence on molecular behavior.

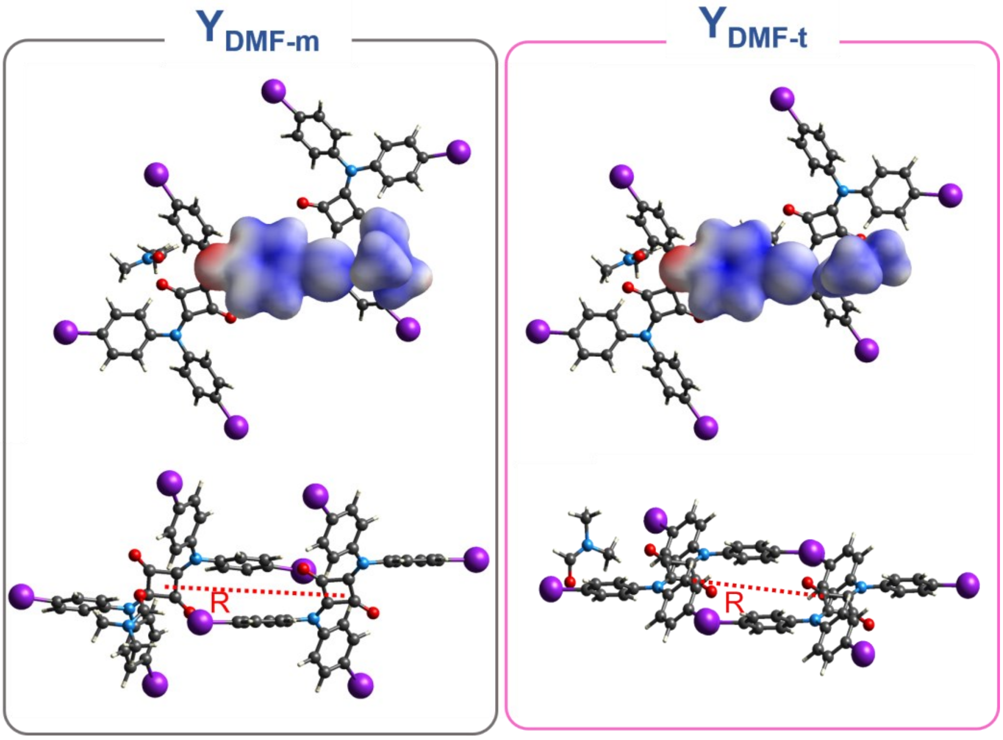

	R	E_{ele}	E_{pol}	E_{int}
Y_{DMF-m}	9.80	-47.6	-6.7	-67.0
Y_{DMF-t}	10.16	-53.7	-5.8	-68.5

Figure S40. Employed CE-B3LYP/def2-TZVP (CrystalExplorer).[23] Interaction Energies (kJ/mol): The following energy components are detailed: E_{ele}- Electrostatic Energy, E_{pol}- Polarization Energy, and E_{int}- Interaction Energy. 'R' indicates the distance between the centers of mass of the molecules, measured in Angstroms (Å).

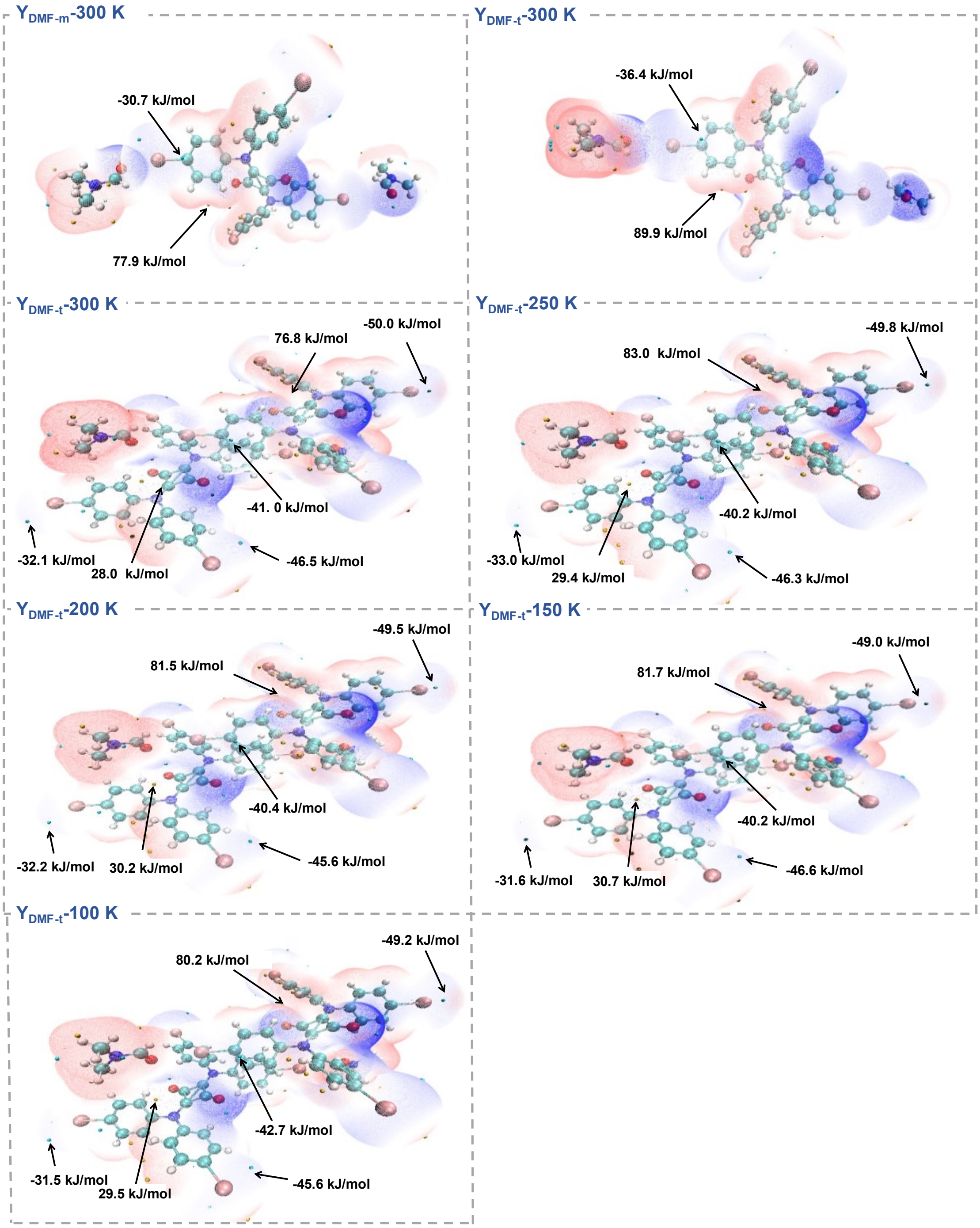
Y_{DMF-m}-300 K
-30.7 kJ/mol
77.9 kJ/mol
Y_{DMF-t}-300 K
-36.4 kJ/mol
89.9 kJ/mol
Y_{DMF-t}-300 K
-50.0 kJ/mol
76.8 kJ/mol
-41. 0 kJ/mol
-32.1 kJ/mol
28.0 kJ/mol
-46.5 kJ/mol
Y_{DMF-t}-250 K
-49.8 kJ/mol
83.0 kJ/mol
-40.2 kJ/mol
-33.0 kJ/mol
29.4 kJ/mol
-46.3 kJ/mol
Y_{DMF-t}-200 K
-49.5 kJ/mol
81.5 kJ/mol
-40.4 kJ/mol
-32.2 kJ/mol
30.2 kJ/mol
-45.6 kJ/mol
Y_{DMF-t}-150 K
-49.0 kJ/mol
81.7 kJ/mol
-40.2 kJ/mol
-31.6 kJ/mol
30.7 kJ/mol
-46.6 kJ/mol
Y_{DMF-t}-100 K
-49.2 kJ/mol
80.2 kJ/mol
-42.7 kJ/mol
-31.5 kJ/mol
29.5 kJ/mol
-45.6 kJ/mol

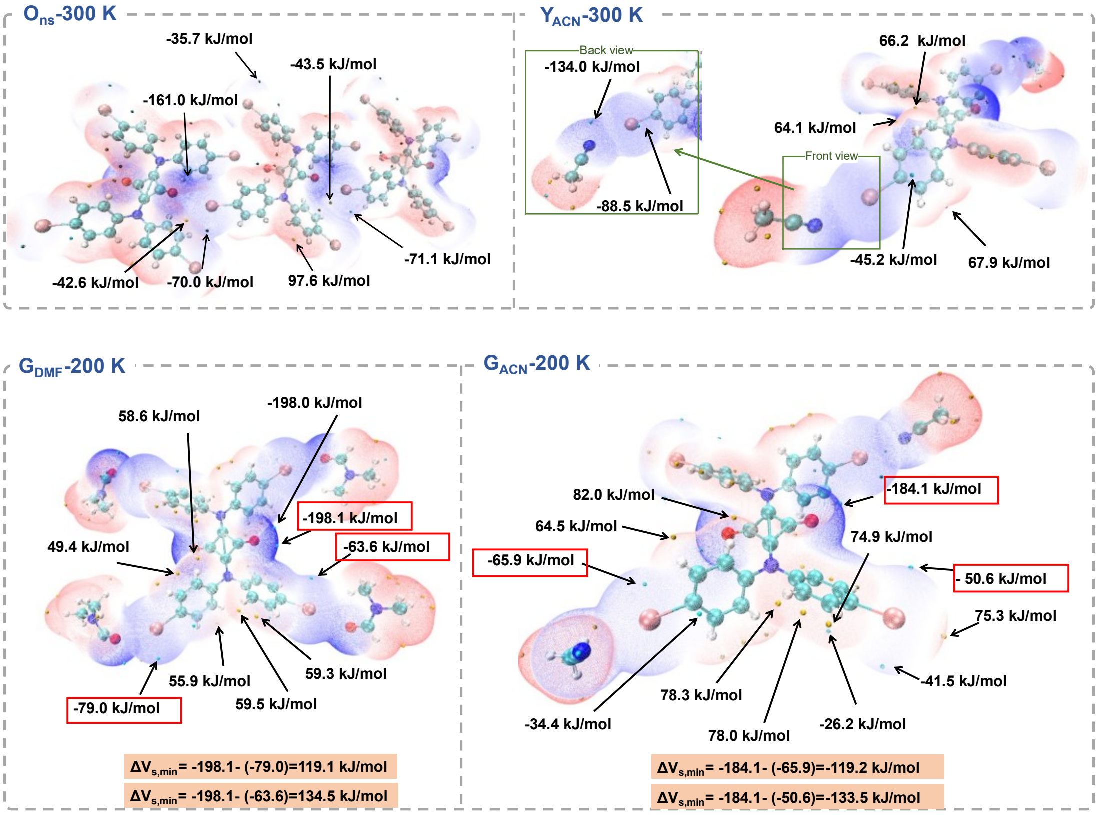

Figure S41. The ESP mapped molecular vdW surface (the isosurface of $\rho = 0.001$ e/bohr^3). calculated for the geometries of SQD-I found in corresponding crystal structures. (Significant surface local minima and maxima of ESP are represented as orange and cyan spheres, and labelled by black texts.) (Value of positive potential in the critical point located above the central area of the N(SQD-I) and aromatic H. Value of negative potential in the critical point located on I.) (We have used Density Functional Theory (DFT) calculations to determine the electrostatic potential maps of SQD-I geometries. These calculations were performed using the Gaussian 16[29] with the B3LYP functional and the DEF2TZVP basis set.[11] The color mapped isosurface graphs of ESP were created using the Multiwfn 3.8 (dev) [26] program, enabling the interpretation of the electrostatic characteristics of the molecules.)

For the SCSC transformation from **G$_{DMF}$** to **G$_{ACN}$**: The term "maintaining the original charge distribution" refers to the consistent ESP arrangement within SQD-I even after the exchange with acetonitrile guests. The difference between the surface local minima values of iodine (I) and oxygen (O) is found to be very similar. For **G$_{DMF}$**, ΔVs,min are 119.1 kJ/mol and 134.5 kJ/mol for I and O, respectively, while for **G$_{ACN}$**, the $\Delta V_{s,min}$ are 119.2 kJ/mol and 133.5 kJ/mol. These results demonstrate that the charge distribution remains consistent, indicating the persistence of the original electrostatic potential pattern in SQD-I even with the introduction of acetonitrile guest molecules.

v. Void space analysis

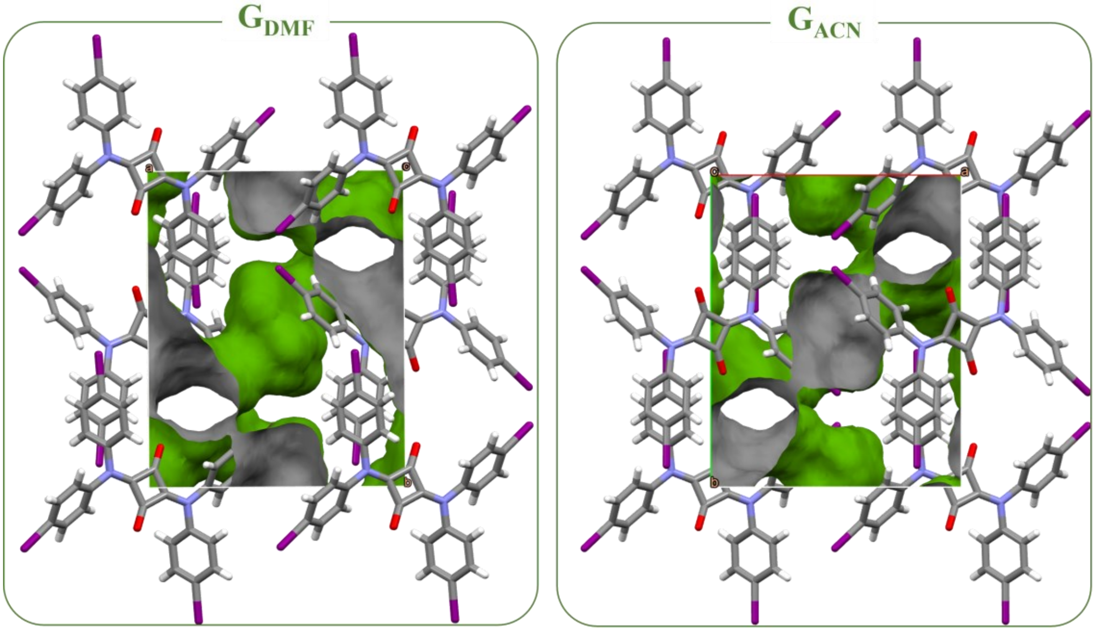

Figure S42. Packing diagrams of **G_{DMF}** and **G_{ACN}** in c-axis, with the solvent-accessible void space visualized by grey/green (inner/outer) curved planes generated with a probe of 1.2 Å. (Volume: **G_{DMF}**: 38.3% of unit cell volume, 775.25 Å^3; **G_{ACN}**: 34.0% of unit cell volume, 631.39 Å^3.)

V. Crystal data and structure refinement table

Table S3. Cell parameters of Y $_{DMF-m}$ from 240 K to 210 K

Cell parameters	Y $_{DMF-m}$-240 K	Y $_{DMF-m}$-230 K	Y $_{DMF-m}$-220 K	Y $_{DMF-m}$-210 K
a/Å	10.9109(4)	10.8979(3)	10.8809(4)	9.9828(9)
b/Å	14.3854(7)	14.3747(5)	14.3553(5)	10.1144(7)
c/Å	12.3998(4)	12.3955(3)	12.3808(4)	10.5874(9)
α/°	90	90	90	116.582(8)
β/°	110.877(4)	110.769(3)	110.624(4)	103.747(8)
γ/°	90	90	90	94.901(7)
Volume/Å^3	1818.47(13)	1815.62(10)	1809.93(11)	905.71(13)

Table S4. Crystal data and structure refinement of crystals

	O_{ns}	Y_{ACN}	G_{DMF}	G_{ACN}
CCDC number	2277097	2277087	2277100	2277093
Empirical formula	$C_{28}H_{16}I_4N_2O_2$	$C_{32}H_{22}I_4N_4O_2$	$C_{37}H_{37}I_4N_5O_5$	$C_{34}H_{25}I_4N_5O_2$
Formula weight	920.03	1002.13	1139.31	1043.19
Temperature	303(2) K	300(2) K	200(2) K	200(2) K
Wavelength	1.54178 Å	1.54178 Å	1.54178 Å	1.54178 Å
Crystal system	Triclinic	Triclinic	Monoclinic	Monoclinic
Space group	*P*−1	*P*−1	$P2_1/c$	$P2_1/c$
Unit cell dimensions	a = 4.2913(2) Å<br>b = 12.0541(5) Å<br>c = 13.8643(8) Å	a = 7.3219(4) Å<br>b = 9.9426(6) Å<br>c = 12.1517(6) Å	a = 13.9680(15) Å<br>b = 16.7251(11) Å<br>c = 8.9232(8) Å	a = 13.8266(17) Å<br>b = 16.4286(12) Å<br>c = 8.5555(10) Å
Volume	696.61(6) Å^3	843.34(8) Å^3	2024.5(3) Å^3	1857.8(4) Å^3
Z	1	1	2	2
Density (calculated)	2.193 Mg/m^3	1.973 Mg/m^3	1.869 Mg/m^3	1.865 Mg/m^3
Absorption coefficient	35.354 mm^{-1}	29.287 mm^{-1}	24.552 mm^{-1}	26.629 mm^{-1}
F(000)	426	470	1092	984
Crystal size	0.100 x 0.100 x 0.050 mm^3	0.100 x 0.100 x 0.050 mm^3	0.100 x 0.100 x 0.020 mm^3	0.100 x 0.100 x 0.020 mm^3
Theta range for data collection	3.234 to 74.297°.	3.784 to 66.575°.	3.258 to 74.435°.	3.344 to 74.445°.
Index ranges	-5≤h≤3,<br>-14≤k≤14,<br>-17≤l≤16	-8≤h≤8,<br>-11≤k≤11,<br>-13≤l≤14	-17≤h≤16,<br>-18≤k≤20,<br>-10≤l≤10	-16≤h≤17,<br>-17≤k≤19,<br>-10≤l≤10

Reflections collected	7688	7865	11993	10181
Independent reflections	2716 [R(int) = 0.0386]	2920 [R(int) = 0.0338]	3944 [R(int) = 0.0479]	3649 [R(int) = 0.0562]
Completeness to theta = 67.679°	99.00%	97.90%	99.60%	99.50%
Absorption correction	Semi-empirical from equivalents	Semi-empirical from equivalents	Semi-empirical from equivalents	Semi-empirical from equivalents
Max. and min. transmission	1.00000 and 0.46541	1.00000 and 0.49787	1.00000 and 0.53983	1.00000 and 0.23604
Refinement method	Full-matrix least-squares on F^2	Full-matrix least-squares on F^2	Full-matrix least-squares on F^2	Full-matrix least-squares on F^2
Data / restraints / parameters	2716 / 0 / 164	2920 / 0 / 192	3944 / 51 / 258	3649 / 6 / 220
Goodness-of-fit on F^2	1.15	1.036	1.036	1.07
Final R indices [I>2sigma(I)]	R1 = 0.0547, wR2 = 0.1397	R1 = 0.0343, wR2 = 0.0955	R1 = 0.0534, wR2 = 0.1016	R1 = 0.0679, wR2 = 0.1822
R indices (all data)	R1 = 0.0737, wR2 = 0.1559	R1 = 0.0400, wR2 = 0.0987	R1 = 0.0812, wR2 = 0.1115	R1 = 0.0830, wR2 = 0.1933
Extinction coefficient	0.0008(2)	0.00107(15)	0.00038(6)	n/a
Largest diff. peak and hole	2.335 and -1.228 e.Å^{-3}	0.654 and -0.826 e.Å^{-3}	1.081 and -1.251 e.Å^{-3}	2.068 and -1.374 e.Å^{-3}

	**Y**$_{\mathbf{DMF\text{-}m}}$-300 K	**Y**$_{\mathbf{DMF\text{-}m}}$-250 K	**Y**$_{\mathbf{DMF\text{-}m}}$-200 K
CCDC number	2277089	2277098	2277086
Empirical formula	$C_{34}H_{30}I_4N_4O_4$	$C_{34}H_{30}I_4N_4O_4$	$C_{34}H_{30}I_4N_4O_4$
Formula weight	1066.22	1066.22	1066.22
Temperature	297(2) K	250(2) K	200(2) K
Wavelength	0.71073 Å	0.71073 Å	0.71073 Å
Crystal system	Monoclinic	Monoclinic	Triclinic
Space group	$P2_1/n$	$P2_1/n$	$P-1$
Unit cell dimensions	a = 11.0008(4) Å	a = 10.9122(4) Å	a = 9.9918(8) Å
	b = 14.4283(5) Å	b = 14.3815(6) Å	b = 10.1035(8) Å
	c = 12.4684(5) Å	c = 12.4067(5) Å	c = 10.5221(8) Å
Volume	1841.95(12) Å^3	1819.07(13) Å^3	899.85(12) Å^3
Z	2	2	1
Density (calculated)	1.922 Mg/m^3	1.947 Mg/m^3	1.968 Mg/m^3

Absorption coefficient	3.424 mm^{-1}	3.468 mm^{-1}	3.505 mm^{-1}
F(000)	1012	1012	506
Crystal size	0.400 x 0.200 x 0.200 mm^3	0.400 x 0.200 x 0.200 mm^3	0.400 x 0.200 x 0.200 mm^3
Theta range for data collection	2.252 to 26.371°	2.257 to 26.372°	2.151 to 26.369°
Index ranges	-13≤h≤13, -18≤k≤18, -15≤l≤15	-13≤h≤13, -17≤k≤17, -15≤l≤15	-12≤h≤12, -12≤k≤12, -13≤l≤13
Reflections collected	43638	42583	21603
Independent reflections	3676 [R(int) = 0.0568]	3614 [R(int) = 0.0581]	3633 [R(int) = 0.0315]
Completeness to theta = 25.242°	97.00%	96.60%	98.60%
Absorption correction	Semi-empirical from equivalents	Semi-empirical from equivalents	Semi-empirical from equivalents
Max. and min. transmission	0.7454 and 0.4757	0.7454 and 0.4724	0.7454 and 0.5281
Refinement method	Full-matrix least-squares on F^2	Full-matrix least-squares on F^2	Full-matrix least-squares on F^2
Data / restraints / parameters	3676 / 112 / 259	3614 / 130 / 259	3633 / 0 / 211
Goodness-of-fit on F^2	1.031	1.049	1.197
Final R indices [I>2sigma(I)]	R1 = 0.0432, wR2 = 0.0947	R1 = 0.0361, wR2 = 0.0781	R1 = 0.0397, wR2 = 0.0949
R indices (all data)	R1 = 0.0473, wR2 = 0.0981	R1 = 0.0382, wR2 = 0.0796	R1 = 0.0434, wR2 = 0.0972
Extinction coefficient	0.0104(14)	0.0120(12)	0.0066(14)
Largest diff. peak and hole	1.576 and -1.695 e.Å^{-3}	1.591 and -1.711 e.Å^{-3}	1.592 and -1.163 e.Å^{-3}

	$\mathbf{Y}_{\mathbf{DMF\text{-}m}}$-100 K	$\mathbf{Y}_{\mathbf{DMF\text{-}m}}$-200 K-RE	$\mathbf{Y}_{\mathbf{DMF\text{-}m}}$-300 K -RE
CCDC number	2277096	2277090	2277094
Empirical formula	$C_{34}H_{30}I_4N_4O_4$	$C_{34}H_{30}I_4N_4O_4$	$C_{34}H_{30}I_4N_4O_4$
Formula weight	1066.22	1066.22	1066.22
Temperature	108(2) K	200(2) K	300(2) K
Wavelength	0.71073 Å	0.71073 Å	0.71073 Å
Crystal system	Triclinic	Triclinic	Triclinic
Space group	*P*−1	*P*−1	*P*−1
Unit cell dimensions	a = 9.9975(9) Å	a = 9.9873(6) Å	a = 9.9606(8) Å

	b = 10.0883(9) Å	b = 10.1030(6) Å	b = 10.1339(7) Å
	c = 10.4290(9) Å	c = 10.5187(6) Å	c = 10.7451(8) Å
Volume	888.32(14) $Å^3$	898.95(9) $Å^3$	922.76(12) $Å^3$
Z	1	1	1
Density (calculated)	1.993 Mg/m^3	1.970 Mg/m^3	1.919 Mg/m^3
Absorption coefficient	3.550 mm^{-1}	3.508 mm^{-1}	3.418 mm^{-1}
F(000)	506	506	506
Crystal size	0.400 x 0.200 x 0.200 mm^3	0.400 x 0.200 x 0.200 mm^3	0.400 x 0.200 x 0.200 mm^3
Theta range for data collection	2.150 to 26.409°.	2.153 to 26.360°.	2.163 to 26.407°.
Index ranges	-12≤h≤12, -12≤k≤12, -12≤l≤13	-12≤h≤12, -12≤k≤12, -13≤l≤13	-12≤h≤12, -12≤k≤12, -13≤l≤13
Reflections collected	14917	21707	21906
Independent reflections	3569 [R(int) = 0.0277]	3618 [R(int) = 0.0292]	3751 [R(int) = 0.0389]
Completeness to theta = 25.242°	97.80%	98.20%	98.90%
Absorption correction	Semi-empirical from equivalents	Semi-empirical from equivalents	Semi-empirical from equivalents
Max. and min. transmission	0.7454 and 0.5594	0.7454 and 0.5419	0.7454 and 0.5194
Refinement method	Full-matrix least-squares on F^2	Full-matrix least-squares on F^2	Full-matrix least-squares on F^2
Data / restraints / parameters	3569 / 0 / 211	3618 / 0 / 211	3751 / 51 / 210
Goodness-of-fit on F^2	1.277	1.16	1.037
Final R indices [I>2sigma(I)]	R1 = 0.0379, wR2 = 0.1009	R1 = 0.0381, wR2 = 0.0875	R1 = 0.0617, wR2 = 0.1489
R indices (all data)	R1 = 0.0395, wR2 = 0.1017	R1 = 0.0414, wR2 = 0.0896	R1 = 0.0708, wR2 = 0.1565
Extinction coefficient	0.0118(17)	0.0054(13)	n/a
Largest diff. peak and hole	2.166 and -1.164 e.$Å^{-3}$	1.615 and -1.260 e.$Å^{-3}$	3.216 and -2.345 e.$Å^{-3}$

	$\mathbf{Y_{DMF-t}}$-300 K	$\mathbf{Y_{DMF-t}}$-250 K	$\mathbf{Y_{DMF-t}}$-200 K
	2277095	2277088	2277091
Empirical formula	$C_{34}H_{30}I_4N_4O_4$	$C_{34}H_{30}I_4N_4O_4$	$C_{34}H_{30}I_4N_4O_4$
Formula weight	1066.22	1066.22	1066.22

Temperature	300(2) K	250(2) K	200(2) K
Wavelength	1.54178 Å	1.54178 Å	1.54178 Å
Crystal system	Triclinic	Triclinic	Triclinic
Space group	*P*−1	*P*−1	*P*−1
Unit cell dimensions	a = 9.9675(4) Å	a = 9.9946(2) Å	a = 10.0015(3) Å
	b = 10.1596(4) Å	b = 10.1375(2) Å	b = 10.1205(4) Å
	c = 10.7649(4) Å	c = 10.6227(3) Å	c = 10.5358(4) Å
Volume	927.31(7) $Å^3$	913.66(4) $Å^3$	903.27(6) $Å^3$
Z	1	1	1
Density (calculated)	1.909 Mg/m^3	1.938 Mg/m^3	1.960 Mg/m^3
Absorption coefficient	26.721 mm^{-1}	27.120 mm^{-1}	27.432 mm^{-1}
F(000)	506	506	506
Crystal size	0.200 x 0.200 x 0.100 mm^3	0.200 x 0.200 x 0.100 mm^3	0.100 x 0.050 x 0.030 mm^3
Theta range for data collection	4.694 to 74.439°.	4.674 to 66.589°.	4.667 to 74.445°.
Index ranges	-12≤h≤12, -12≤k≤9, -12≤l≤13	-11≤h≤11, -12≤k≤9, -12≤l≤12	-12≤h≤12, -12≤k≤9, -12≤l≤13
Reflections collected	9877	9093	9733
Independent reflections	3596 [R(int) = 0.0627]	3174 [R(int) = 0.0820]	3521 [R(int) = 0.0789]
Completeness to theta = 67.679°	98.80%	98.70%	98.60%
Absorption correction	Semi-empirical from equivalents	Semi-empirical from equivalents	Semi-empirical from equivalents
Max. and min. transmission	1.00000 and 0.38358	1.00000 and 0.45537	1.00000 and 0.41532
Refinement method	Full-matrix least-squares on F^2	Full-matrix least-squares on F^2	Full-matrix least-squares on F^2
Data / restraints / parameters	3596 / 0 / 211	3174 / 0 / 211	3521 / 0 / 211
Goodness-of-fit on F^2	1.125	1.152	1.149
Final R indices [I>2sigma(I)]	R1 = 0.0666, wR2 = 0.1838	R1 = 0.0576, wR2 = 0.1644	R1 = 0.0487, wR2 = 0.1397
R indices (all data)	R1 = 0.0733, wR2 = 0.1909	R1 = 0.0612, wR2 = 0.1684	R1 = 0.0510, wR2 = 0.1423
Extinction coefficient	0.0039(4)	0.0028(4)	0.0025(3)

Largest diff. peak and hole	3.030 and -1.869 e.Å^{-3}	2.402 and -2.089 e.Å^{-3}	2.532 and -1.441 e.Å^{-3}

	**Y$_{\mathbf{DMF\text{-}t}}$**-150 K	**Y$_{\mathbf{DMF\text{-}t}}$**-100 K	**Y$_{\mathbf{DMF\text{-}t}}$**-150 K-RE
	2277101	2277085	2277102
Empirical formula	$C_{34}H_{30}I_4N_4O_4$	$C_{34}H_{30}I_4N_4O_4$	$C_{34}H_{30}I_4N_4O_4$
Formula weight	1066.22	1066.22	1066.22
Temperature	150(2) K	102(2) K	150(2) K
Wavelength	1.54178 Å	1.54178 Å	1.54178 Å
Crystal system	Triclinic	Triclinic	Triclinic
Space group	*P*−1	*P*−1	*P*−1
Unit cell dimensions	a = 10.0033(3) Å	a = 10.0024(3) Å	a = 10.0041(4) Å
	b = 10.1063(4) Å	b = 10.0944(4) Å	b = 10.1072(4) Å
	c = 10.4768(4) Å	c = 10.4267(3) Å	c = 10.4788(4) Å
Volume	895.75(6) Å^3	888.99(6) Å^3	896.10(7) Å^3
Z	1	1	1
Density (calculated)	1.977 Mg/m^3	1.992 Mg/m^3	1.976 Mg/m^3
Absorption coefficient	27.662 mm^{-1}	27.873 mm^{-1}	27.651 mm^{-1}
F(000)	506	506	506
Crystal size	0.200 x 0.100 x 0.100 mm^3	0.200 x 0.100 x 0.100 mm^3	0.200 x 0.100 x 0.100 mm^3
Theta range for data collection	4.665 to 74.410°.	4.666 to 74.405°.	4.665 to 74.375°.
Index ranges	-12≤h≤12, -12≤k≤9, -12≤l≤13	-12≤h≤12, -12≤k≤9, -12≤l≤13	-12≤h≤12, -12≤k≤9, -12≤l≤13
Reflections collected	9147	9064	9325
Independent reflections	3491 [R(int) = 0.0662]	3455 [R(int) = 0.0699]	3473 [R(int) = 0.0684]
Completeness to theta = 67.679°	98.50%	98.40%	98.50%
Absorption correction	Semi-empirical from equivalents	Semi-empirical from equivalents	Semi-empirical from equivalents
Max. and min. transmission	1.00000 and 0.40795	1.00000 and 0.31345	1.00000 and 0.28605
Refinement method	Full-matrix least-squares on F^2	Full-matrix least-squares on F^2	Full-matrix least-squares on F^2
Data / restraints / parameters	3491 / 0 / 211	3455 / 0 / 191	3473 / 0 / 191
Goodness-of-fit on F^2	1.063	1.032	1.112

Final R indices [I>2sigma(I)]	R1 = 0.0427, wR2 = 0.1158	R1 = 0.0404, wR2 = 0.1101	R1 = 0.0539, wR2 = 0.1568
R indices (all data)	R1 = 0.0443, wR2 = 0.1175	R1 = 0.0416, wR2 = 0.1115	R1 = 0.0555, wR2 = 0.1589
Extinction coefficient	0.0015(2)	0.00146(18)	0.0013(3)
Largest diff. peak and hole	2.291 and -1.589 e.Å^{-3}	1.664 and -1.487 e.Å^{-3}	3.189 and -1.825 e.Å^{-3}

	$\mathbf{Y_{DMF\text{-}t}}$-200 K-RE	$\mathbf{Y_{DMF\text{-}t}}$-250 K-RE	$\mathbf{Y_{DMF\text{-}t}}$-300 K-RE
CCDC number	2277103	2277092	2277099
Empirical formula	$C_{34}H_{30}I_4N_4O_4$	$C_{34}H_{30}I_4N_4O_4$	$C_{34}H_{30}I_4N_4O_4$
Formula weight	1066.22	1066.22	1066.22
Temperature	200(10) K	250(2) K	300(2) K
Wavelength	1.54178 Å	1.54178 Å	1.54178 Å
Crystal system	Triclinic	Triclinic	Triclinic
Space group	*P*−1	*P*−1	*P*−1
Unit cell dimensions	a = 10.0020(2) Å	a = 9.9919(2) Å	a = 9.9635(3) Å
	b = 10.1250(2) Å	b = 10.1388(2) Å	b = 10.1485(3) Å
	c = 10.5425(2) Å	c = 10.6326(3) Å	c = 10.7693(4) Å
Volume	904.41(3) Å^3	914.48(4) Å^3	927.19(6) Å^3
Z	1	1	1
Density (calculated)	1.958 Mg/m^3	1.936 Mg/m^3	1.910 Mg/m^3
Absorption coefficient	27.397 mm^{-1}	27.096 mm^{-1}	26.724 mm^{-1}
F(000)	506	506	506
Crystal size	0.200 x 0.100 x 0.100 mm^3	0.200 x 0.100 x 0.100 mm^3	0.200 x 0.100 x 0.100 mm^3
Theta range for data collection	4.668 to 74.491°.	4.676 to 66.555°.	4.696 to 74.461°.
Index ranges	-12≤h≤12, -12≤k≤9, -12≤l≤13	-11≤h≤11, -12≤k≤9, -12≤l≤12	-12≤h≤12, -12≤k≤9, -12≤l≤13
Reflections collected	9217	8715	9759
Independent reflections	3485 [R(int) = 0.0752]	3173 [R(int) = 0.0737]	3594 [R(int) = 0.0617]
Completeness to theta = 67.679°	98.30%	98.60%	98.40%
Absorption correction	Semi-empirical from equivalents	Semi-empirical from equivalents	Semi-empirical from equivalents
Max. and min. transmission	1.00000 and 0.29688	1.00000 and 0.25371	1.00000 and 0.37035

Refinement method	Full-matrix least-squares on F^2	Full-matrix least-squares on F^2	Full-matrix least-squares on F^2
Data / restraints / parameters	3485 / 0 / 206	3173 / 0 / 211	3594 / 0 / 211
Goodness-of-fit on F^2	1.12	1.123	1.117
Final R indices [I>2sigma(I)]	R1 = 0.0487, wR2 = 0.1345	R1 = 0.0553, wR2 = 0.1553	R1 = 0.0712, wR2 = 0.1953
R indices (all data)	R1 = 0.0511, wR2 = 0.1372	R1 = 0.0597, wR2 = 0.1612	R1 = 0.0795, wR2 = 0.2037
Extinction coefficient	0.0021(3)	0.0019(3)	0.0018(3)
Largest diff. peak and hole	2.302 and -1.795 e.Å^{-3}	2.943 and -1.914 e.Å^{-3}	2.967 and -2.069 e.Å^{-3}

VI. References

[17] G. Oszlanyi, A. Suto, *Acta Crystallogr. A*, **2004**, *60*, 134–141.

[18] G. Sheldrick, *Acta Crystallogr. A*, **2015**, *71*, 3–8.

[19] G. Sheldrick, *Acta Crystallogr. C*, **2015**, *71*, 3–8.

[20] Y. Du, Y. Zhu, S. Xi, P. Yang, H. O. Moser, M. B. H. Breese, A. Borgna, *J. Synchrotron Rad.* **2015**, *22*, 839–843.

[21] B. Ravel, M. Newville, *J. Synchrotron Rad.* **2005**, *12*, 537–541.

[22] Y.-S. Lin, G.-D. Li, S.-P. Mao, J.-D. Chai, *J. Chem. Theory Comput.* **2013**, *9*, 263–272.

[23] S. P. Thomas, P. R. Spackman, D. Jayatilaka, M. A. Spackman, *J. Chem. Theory Comput.* **2018**, *14*, 1614−1623;

[24] F. Neese, *WIREs Comput. Mol. Sci.* **2012**, *2*, 73–78.

[25] E. R. Johnson, S. Keinan, P. Mori-Sánchez, J. Contreras-García, A. J. Cohen, W. Yang, *J. Am. Chem. Soc.* **2010**, *132*, 6498–6506.

[26] T. Lu, F. Chen, *J. Comput. Chem.* **2012**, *33*, 580–592.

[27] W. Humphrey, A. Dalke, K. Schulten, *J. Mol. Graph.* **1996**, *14*, 33–38.

[28] P. R. Spackman, M. J. Turner, J. J. McKinnon, S. K. Wolff, D. J. Grimwood, D. Jayatilaka, M. A. Spackman, *J. Appl. Crystallogr.* **2021**, *54*, 1006-1011.

[29] Gaussian 16, Revision A.03, M. J. Frisch, G. W. Trucks, H. B. Schlegel, G. E. Scuseria, M. A. Robb, J. R. Cheeseman, G. Scalmani, V. Barone, G. A. Petersson, H. Nakatsuji, X. Li, M. Caricato, A. V. Marenich, J. Bloino, B. G. Janesko, R. Gomperts, B. Mennucci, H. P. Hratchian, J. V. Ortiz, A. F. Izmaylov, J. L. Sonnenberg, D. Williams-Young, F. Ding, F. Lipparini, F. Egidi, J. Goings, B. Peng, A. Petrone, T. Henderson, D. Ranasinghe, V. G. Zakrzewski, J. Gao, N. Rega, G. Zheng, W. Liang, M. Hada, M. Ehara, K. Toyota, R. Fukuda, J. Hasegawa, M. Ishida, T. Nakajima, Y. Honda, O. Kitao, H. Nakai, T. Vreven, K. Throssell, J. A. Montgomery, Jr., J. E. Peralta, F. Ogliaro, M. J. Bearpark, J. J. Heyd, E. N. Brothers, K. N. Kudin, V. N. Staroverov, T. A. Keith, R. Kobayashi, J. Normand, K. Raghavachari, A. P. Rendell, J. C. Burant, S. S. Iyengar, J. Tomasi, M. Cossi, J. M. Millam, M. Klene, C. Adamo, R. Cammi, J. W. Ochterski, R. L. Martin, K. Morokuma, O. Farkas, J. B. Foresman, and D. J. Fox, Gaussian, Inc., Wallingford CT, 2016.
